# Recent developments in the design and synthesis of benzylpyridinium salts: Mimicking donepezil hydrochloride in the treatment of Alzheimer’s disease

**DOI:** 10.3389/fchem.2022.936240

**Published:** 2022-09-26

**Authors:** Saghi Sepehri, Mina Saeedi, Bagher Larijani, Mohammad Mahdavi

**Affiliations:** ^1^ Department of Medicinal Chemistry, School of Pharmacy, Ardabil University of Medical Sciences, Ardabil, Iran; ^2^ Pharmaceutical Sciences Research Center, Ardabil University of Medical Sciences, Ardabil, Iran; ^3^ Medicinal Plants Research Center, Faculty of Pharmacy, Tehran University of Medical Sciences, Tehran, Iran; ^4^ Persian Medicine and Pharmacy Research Center, Tehran University of Medical Sciences, Tehran, Iran; ^5^ Endocrinology and Metabolism Research Center, Endocrinology and Metabolism Research Institute, Tehran University of Medical Sciences, Tehran, Iran

**Keywords:** Alzheimer’s disease, acetylcholinesterase, butyrylcholine esterase, donepezil, cholinesterase, benzylpyridinium salts

## Abstract

**Background:** Alzheimer’s disease (AD) is an advanced and irreversible degenerative disease of the brain, recognized as the key reason for dementia among elderly people. The disease is related to the reduced level of acetylcholine (ACh) in the brain that interferes with memory, learning, emotional, and behavior responses. Deficits in cholinergic neurotransmission are responsible for the creation and progression of numerous neurochemical and neurological illnesses such as AD.

**Aim:** Herein, focusing on the fact that benzylpyridinium salts mimic the structure of donepezil hydrochlorideas a FDA-approved drug in the treatment of AD, their synthetic approaches and inhibitory activity against cholinesterases (ChEs) were discussed. Also, molecular docking results and structure–activity relationship (SAR) as the most significant concept in drug design and development were considered to introduce potential lead compounds. *Key scientific concepts:* AChE plays a chief role in the end of nerve impulse transmission at the cholinergic synapses. In this respect, the inhibition of AChE has been recognized as a key factor in the treatment of AD, Parkinson’s disease, senile dementia, myasthenia gravis, and ataxia. A few drugs such as donepezil hydrochloride are prescribed for the improvement of cognitive dysfunction and memory loss caused by AD. Donepezil hydrochloride is a piperidine-containing compound, identified as a well-known member of the second generation of AChE inhibitors. It was established to treat AD when it was assumed that the disease is associated with a central cholinergic loss in the early 1980s. In this review, synthesis and anti-ChE activity of a library of benzylpyridinium salts were reported and discussed based on SAR studies looking for the most potent substituents and moieties, which are responsible for inducing the desired activity even more potent than donepezil. It was found that linking heterocyclic moieties to the benzylpyridinium salts leads to the potent ChE inhibitors. In this respect, this review focused on the recent reports on benzylpyridinium salts and addressed the structural features and SARs to get an in-depth understanding of the potential of this biologically improved scaffold in the drug discovery of AD.

## Introduction

More than 100 years ago, Alzheimer’s disease (AD) was exposed by Alois Alzheimer in 1907. Despite a lot of effort in the treatment of the disease, it has emerged as a crucial public health issue in the 21st century due to the lack of effective and no clinically accepted therapeutic approach, as well as due to a huge economic burden on the society (Prince et al., 2013). AD is known as an irreversible chronic neurodegenerative disturbance of the CNS start-up on a continuing loss of cognitive skill (Goyal et al., 2017). It generally occurs in the elderly population; however, it appears as an autosomal dominant trait in families in 1%–2% of cases (Bekris et al., 2010). AD is usually characterized by the loss of short-term memory, disorientation, impairment of judgment and reasoning and decision making, language, and learning. Patients misplace their capability to connect, fail to identify their near and dear ones, and become bedridden at the last steps of the disease (Sharma, 2019).

The pathogenesis of AD has not yet been definitely clarified. It is extensively recognized that a mixture of environmental activities and genetic vulnerability features is responsible for outspread late-onset AD. Comprehending the mechanism of creation of the disease has been remained as a key factor for developing effective anti-AD drugs. AD is a multifactorial disease in which the formation of toxic amyloid beta (Aβ) (Matsuzaki, 2011), tau protein hyperphosphorylation (Miao et al., 2019), neuroinflammation (Calsolaro and Edison, 2016), oxidative stress (Tonnies and Trushina, 2017), and biometals (Lavado et al., 2019) play vital aspects in the creation and progression of illness. Also, it has been extensively assumed that low levels of acetylcholine (ACh) play a crucial role in the creation of AD (McGleenon et al., 1999; Lan et al., 2017a).

Now, there are a few U.S. FDA-approved drugs, which are prescribed to the patients with AD that only relieve the symptoms of AD. Among them, donepezil, galantamine, and rivastigmine are cholinesterase (ChE) inhibitors. They avoid the hydrolysis of acetylcholine (ACh), which is the main neurotransmitter of the parasympathetic nervous system in the brain and responsible for learning and memory (Picciotto et al., 2012). It should be noted that tacrine was also the approved drug in the same category, which was removed from the market due to hepatotoxicity (Girek and Szymanski, 2019). Another drug is memantine belonging to different classes of anti-AD agents, NMDAR antagonists (Mangialasche et al., 2010). However, the combination of donepezil and memantine is currently used to improve physical and mental health in patients with AD (Wake et al., 2018). As mentioned above, all these drugs do not treat the disease definitely and relieve its signs to increase the quality of life of patients and caregivers. In this regard, the design and development of anti-AD drugs is in urgent demand (Lee and Kim, 2017).

The cholinergic hypothesis is the most general description of the mechanism of AD progression, which directly contributes to the cognitive decline (Bartus, 2000). Amyloid protein plaques can be created using both ChEs, AChE and butyrylcholinesterase (BuChE), in which their inhibitors can reduce them (Yu L. et al., 2010). BuChE is generally of glial origin, while AChE is typically of neuronal origin (Mesulam et al., 2002). Under usual conditions, ACh is frequently disintegrated using AChE instead of BuChE (Mack and Robitzki, 2000).

As the decline of ACh levels in the hippocampus and cortex leads to reasoning and memory shortfalls, refining cholinergic function has been completely measured in the treatment of AD (Stanciu et al., 2020). Several studies have also indicated that AChE seems to be implicated in the pathogenesis of AD by promoting the formation of both Aβ fibrils. Fortunately, AChEIs possibly affect the metabolic processing of the amyloid precursor protein (APP) and thus may influence the generation of Aβ (Garcia-Ayllon et al., 2011). Using BuChE, acetylcholine can be hydrolyzed and the levels of AChE can be compensated when they are decreased. In the brain affected by AD with variations becoming more pronounced during the disease course, there is a reduction in AChE levels, whereas BuChE levels are obviously unchanged or increased. Moreover, the BuChE genotype can affect AD risk and the rate of illness development (Nordberg et al., 2013).

It is worth emphasizing that although many hypotheses and strategies are currently being proposed for the treatment of AD, ChEIs still remain to be a supreme clinical success in the treatment of AD, which fully demonstrates the value of this target (Nadri et al., 2010; Jiang et al., 2018).

ChEs have very similar structures. Both of them comprise a deep gorge and a peripheral anionic site (PAS) and a catalytic active site (CAS). Amino acid sequences in the AChE and BuChE are almost 65% homologic (Nicolet et al., 2003). Catalytic triads of human AChE (hAChE) and human BuChE (hBuChE) contain preserved residues: His438, Glu325, and Ser198 in hBuChE, and Glu334, His447, and Ser203 in hAChE (Shafferman et al., 1992). Yet, the existence and number of amino acids inside the gorge are diverse, particularly exhibited using the acyl-binding pocket, which includes an acyl moiety to catalyze the substrate (Brus et al., 2014). There are Phe295 and Phe297 residues in the hAChE pocket, whereas Leu286 and Val288 are in the hBuChE pocket. Two aromatic amino acids of hAChE bulging into the gorge somewhat occupy the space, while the presence of smaller amino acids in hBuChE prepares an extensive space and permits bigger substrates to bind to be hydrolyzed (Li et al., 2017). Diverse structural characteristics of the both enzymes contribute to their substrate particularity: Small molecules such as ACh have a higher affinity toward AChE, whereas numerous peptides have more selectivity for BuChE (Taylor and Radic, 1994). Based on studies, the benzylic moiety of donepezil interacts with the CAS and dimethoxyindanone moiety interacts with the PAS of the AChE (Mohsin and Ahmad, 2020).

**FIGURE 1:**
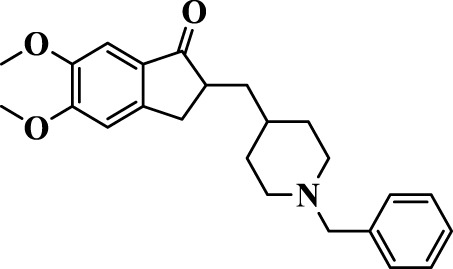
Structure of donepezil.

Donepezil (donepezil hydrochloride, under the brand name Aricept) is a medication used for the symptomatic treatment of mild-to-moderate AD, which was introduced in 1996 (Figure 1). It is a non-competitive and reversible inhibitor of AChE, thus inhibiting ACh hydrolysis. Donepezil appears to compensate for the loss of functioning cholinergic neurons through maintaining high ACh levels (Knowles, 2006). It has demonstrated a 1000-fold selectivity for the inhibition of AChE over BuChE in *in vitro* experiments (Mushtaq et al., 2014). Other mechanisms have been also postulated for the anti-AD activity of donepezil. It has an impact on nicotinic receptors (Cacabelos, 2007) and reduces the decrease in expression in cerebral cortex and avoids the diminution in nicotinic binding that is related to illness harshness (Wu et al., 2010). It declines glutamate neurotoxicity and inhibits excitotoxic damage to keep neuroprotective actions (Shen et al., 2010). As for another important characteristics of AD pathology, oxidative stress has stimulated a great attention. Donepezil has been potentially established to fight against free radicals and improve the effects of oxidative stress in a streptozotocin-induced model of AD mice (Li et al., 2018). The brain AChE is inhibited by the oral administration of this drug in a dose-dependent manner. It has revealed impartially good permeability to the brain. The concentration of donepezil in the brain is about sixfold to sevenfold more than that in plasma. It is a group of ChE inhibitor bearing indanone and *N*-benzylpiperidine moieties that displays longer and more selective action.

**SCHEME 1:**
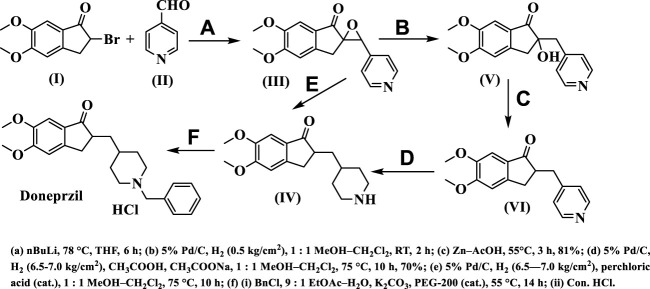
Synthesis procedure of donepezil.

Donepezil or donepezil hydrochloride is being synthesized from arylidene-2-indanone or alkylidene provided using Aldol condensation as crucial intermediates followed by the catalytic reduction (Dubey et al., 2010; Rawat et al., 2013; Costanzo et al., 2016) (Scheme 1).

Based on molecular docking studies, it was found that the dimethoxyindanone moiety is responsible for binding to the peripheral anionic site (PAS) of the AChE *via* aromatic π–π stacking interactions with Trp279, Arg289, Ser286, Phe331, and Tyr121, while the piperidine ring interacts with amino acids Tyr337 and Tyr334 located in the anionic part of catalytic active site (Figure 1). The benzyl moiety of donepezil is in nearby Trp86, Gly118, and Trp84; and Phe330, His447, Glu199, His440, and Ser203 amino acids, both parts of the catalytic triad (Figure 1). Previously, it has been revealed that the modification of the benzyl ring can lead to a strong AChE inhibition (Kryger et al., 1999; Makarian et al., 2022).

As anti-AD activity of donepezil has been proven from various mechanistic points of view, it has been considered as the main scaffold in the design and synthesis of a varied range of compounds against AD (Sugimoto et al., 2002; Agatonovic-Kustrin et al., 2018; Piemonyese et al., 2018; Mohsin and Ahmad, 2020). Herein, we focused on the benzylpyridinium salts, which have shown very good ChE inhibitory activity mimicking the structure of donepezil.

### Coumarin hybrids

**FIGURE 2:**
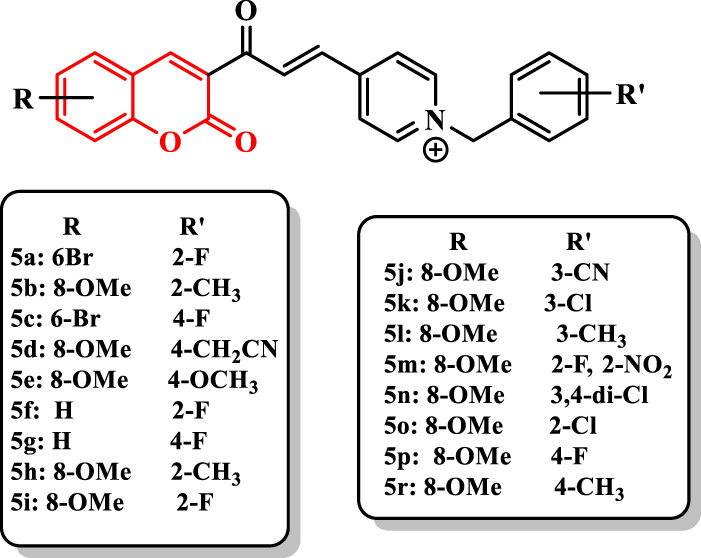
Coumarin derivatives bearing *N*-benzylpyridinium moiety.

Alipour et al. (2012) reported the synthesis of coumarin derivatives bearing *N*-benzylpyridinium moiety (Supplementary Scheme S1). Among the synthesized compounds, **5a** (IC_50_ = 0.11 nM) exhibited higher activity than donepezil (IC_50_ = 14 nM). The compound **5b** exhibited the high selectivity for AChE. Based on results, the introduction of substituents into 2-, 3-, and 4-positions of the benzyl moiety influenced the activity and selectivity (**5a** (IC_50_ = 0.11 nM) vs*.* **5c** (IC_50_ = 0.46 nM)). The existence of the group at 2- and 4-positions showed the highest and lowest anti-AChE activity, respectively, while the nature of the group (electron-withdrawing and electron-donating) was not important for the inhibitory activity. Moreover, the presence of two substituents on the benzyl moiety drastically reduced the activity (**5m**, IC_50_ = 330 nM; **5n**, IC_50_ = 440 nM). In addition, the size of the substituent at 2- and 4-positions [**5d** (IC_50_ = 1,600 nM) and **5e** (IC_50_ = 1,470 nM); (**5h**, IC_50_ = 0.16 nM) vs. (**5i**, IC_50_ = 26 nM)] of compounds is important for the activity. Moreover, according to the data, the presence of more electron-withdrawing substituent at the 3-position of the benzyl moiety of derivatives revealed higher activity in the order of **5b** (IC_50_ = 0.47 nM) > **5j** (IC_50_ = 76 nM) > **5k** (IC_50_ = 86 nM) > **5l** (IC_50_ = 800 nM) (Figure 2).

The same research group (Alipour et al., 2013) synthesized a series of derivatives with coumarin and 3-coumaranone scaffolds connecting to phenacylpyridinium moiety as ChE inhibitors (Supplementary Schemes S2, S3).

All screened compounds showed weaker activity than donepezil as a standard drug (IC_50_ = 0.014 and 5.38 µM against ChEs). Among the synthesized compounds, **16a** showed the most potent activity (IC_50_ = 1.3 µM) against AChE, while it had lower activity than donepezil. According to results, 3-coumaranone derivatives were generally more potent and selective than coumarin derivatives against AChE. By contrast, derivatives unsubstituted at the benzyl moiety displayed the same activities (compounds **9a** and **16b** with IC_50_ values of 10 and 7.4 µM, respectively). Thus, the substituent on the benzyl moiety plays a key role in activity (Supplementary Figure S1).

Khoobi et al. (2013) reported a series of coumarins connected to *N*-benzylpyridinium moiety as ACh inhibitors (Supplementary Scheme S4).

The evaluation of 4-pyridinium derivatives against AChE demonstrated that derivative **21a** (IC_50_ = 0.038 µM) was the most potent compound, while 2- or 3-flouro derivatives (**21b** (IC_50_ = 2.9 µM) and **21c** (IC_50_ = 2.8 µM)) showed lower activity in analogs bearing halides. Furthermore, the presence of 3-chloro, 2,4-dichloro or 3,4-dichloro in the *N*-benzyl group of the 4-pyridinium series enhanced the anti-AChE activity as detected in compounds **21d** (IC_50_ = 0.48 µM), **21e** (IC_50_ = 0.044 µM), and **21f** (IC_50_ = 1.8 µM). Comparing the IC_50_ values of 2- or 3-chloro compounds **21g** (IC_50_ = 1.5 µM) and **21d** (IC_50_ = 0.48 µM) with their 2- or 3-fluoro analogs **21b** and **21c** revealed that the chlorine substituent is more effective than fluorine at 2- and 3-positions.

In the 3-pyridinium series, the fluorine-substituted analogs **21h** (IC_50_ = 1.8 µM), **21i** (IC_50_ = 2.0 µM), and **21j** (IC_50_ = 2.17 µM) showed approximately similar inhibitory activity on AChE. It was found that the fluorine substituent on the *N*-benzyl group of the 3-pyridinum series had no noteworthy effect on inhibitory activity. Unlike, the chlorine substituent on the benzyl group of derivative **21k** (IC_50_ = 1.0 µM) was significantly more active than 3-chlorobenzyl analog **21l** (IC_50_ = 0.79 µM). All compounds showed less activity than donepezil (IC_50_ = 0.014 µM) (Supplementary Figure S2).

Based on results, the position and halogen element on the benzyl ring can control anti-AChE activity and AChE/BuChE selectivity. Among compounds, the derivative **21a** depicted the highest anti-AChE activity (IC_50_ value = 0.038 µM) and the most AChE/BuChE selectivity (SI > 48).

Arab et al. (2015) designed and synthesized a series of chroman-4-one derivatives containing the *N*-benzylpyridinium moiety (**29a-l**) and evaluated them against AChE (Supplementary Scheme S5).

The compound **29a** showed the most potent anti-AChE activity (IC_50_ = 0.048 µM). All compounds showed weaker activity than donepezil as a standard drug (IC_50_ = 0.022 µM).

Based on results, the alkoxy group at the 7-position as well as the type and position of group on the benzyl moiety are important in the anti-AChE activity. Derivatives having the ethoxy group at the 7-position of chroman-4-one showed higher activity than those having the methoxy group at the same position. Thus, an increase in chain length at the 7-position of chroman-4-one was favorable for the desired inhibitory activity.

**FIGURE 3:**
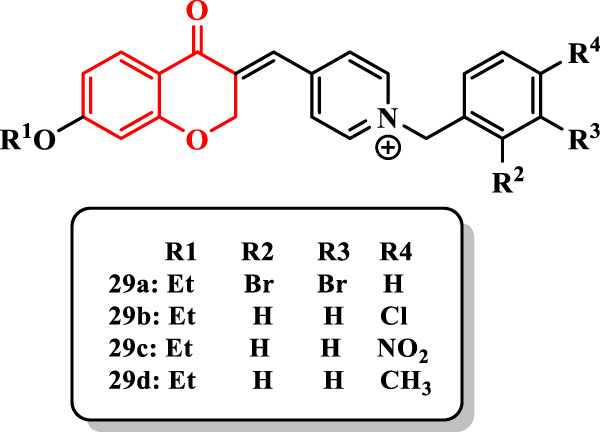
Chroman-4-one derivatives.

Introduction of two bromine substituents into 2- and 3-positions of the benzyl ring (compound **29a**) led to more potent activity than that of one substituent. In addition, the presence of bromine at the 4-position was inappropriate for the activity. Among halides at the 4-position, chlorine showed the highest activity [compound **29b** (IC_50_ = 0.091 µM)], and also, the electron-withdrawing group (NO_2_) was favorable compared with the electron-donating group (CH_3_) [compound **29c** (IC_50_ = 2.713 µM) vs. compound **29d** (IC_50_ = 7.240 µM)] (Figure 3).

Khunnawutmanotham et al. (2016) synthesized some derivatives of scopoletin linked to the pyridinium moiety and investigated them against AChE (Supplementary Scheme S6).

Derivative **33a** showed the highest activity (IC_50_ value of 0.215 µM), which was lower than donepezil (IC_50_ = 0.060 µM). The introduction of chlorine into the 2- and 3-positions of the benzyl group (**33c** and **33d**) showed similar IC_50_ values (0.360 and 0.397 µM, respectively), which were about 15-fold more potent than that of chlorine substituent at the 4-position. Introducing the fluorine into the 2-position dramatically decreased the activity of compounds **33a** and **33b**. In addition, anti-AChE activities of difluorine substituents (**33f** and **33g**) were not remarkably diverse from those of **33a**. Fascinatingly, the IC_50_ value of **33a** was very nearby to that of tacrine; while galantamine exhibited higher activity than the compound **33a** (about 10-fold) (Supplementary Figure S3).

Wang et al. (2015) described a series of 4-isochromanone hybrids as selective AChE inhibitors (Supplementary Scheme S7).

The compound **40a** showed an IC_50_ = 8.9 nM against AChE, in which its inhibitory activity was sixfold greater than that of donepezil (IC_50_ = 59.9 nM). Furthermore, the unsubstituted compound **40b** and halogen-substituted analogs such as **40c** (IC_50_ = 21.1 nM) and **40d** (IC_50_ = 15.9 nM) all revealed good activities against AChE, more potent than donepezil. Particularly, as to the intermediate **39**, missing the benzylpyridinium fragment, a dramatic decrease was observed in the anti-AChE activity (IC_50_ > 100 µM). Thus, it was recommended that this moiety was essential for higher activity.

**FIGURE 6:**
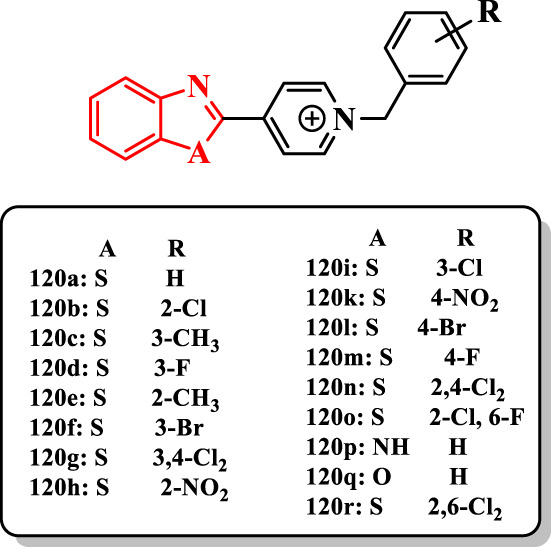
Benzimidazole, benzoxazole, and benzothiazole derivatives.

The introduction of the chlorine and the fluorine groups into 2 (**40c**, IC_50_ = 21.1 nM)-, 3 (**40e**, IC_50_ = 26.3 nM)-, and 4 (**40a**, IC_50_ = 8.93 nM)-positions retains or improves the activity of the corresponding compounds, whereas the replacement of the halide on the benzyl moiety with the nitro or methoxy group dramatically reduced the activity of compounds such as **40f** (IC_50_ = 640 nM) and **40g** (IC_50_ = 503 nM) (Figure 6). Remarkably, the groups at the 4-position had the most influence on the AChE inhibitory activity. Based on results, the presence of the group except for the fluorine at the 4-position of the benzyl moiety decreased the inhibitory activity of the compounds such as **40h** (IC_50_ = 1,193 nM), **40i** (IC_50_ = 646 nM), and **40j** (IC_50_ = 3,548 nM) strangely (Supplementary Figure S4). It was recognized that the small size of fluorine was as similar as hydrogen.

Lan et al. (2017b) reported a series of donepezil derivatives as ChE inhibitors (Supplementary Scheme S8).

The compound **45a** (IC_50_ = 1.9 nM) displayed the highest AChE inhibitory activity. This compound was about 21-fold more potent than donepezil (IC_50_ = 40.2 nM). Fluorine and hydrogen substituents compared with diverse substituents such as NO_2_, Br, and CH_3_ were in attention of maintaining and increasing the anti-AChE activity. For instance, compounds **45b** (IC_50_ = 2.9 nM) and **45c** (IC_50_ = 4.6 nM) with fluorine and hydrogen substituents were more potent than compounds **45d** (IC_50_ = 25.6 nM), **45e** (IC_50_ = 77.3 nM), and **45f** (IC_50_ = 74.6 nM) (Supplementary Figure S4). Also, the presence of substituents at the 2- or 3-position showed higher activity than at the 4-position (compounds **45g** (IC_50_ = 2.9 nM) and **45e)** (Supplementary Figure S5). All tested compounds presented more potent inhibitors for hAChE than for eeAChE. The compound **45a** (IC_50_ = 0.8 nM) showed the most potent inhibition about 47-fold higher than donepezil (IC_50_ = 37.6 nM). The compound **45a** (SI > 5263.1) was the highest selective AChE/BuChE inhibitor.

The same research group (Lan et al., 2017c) designed and synthesized several coumarin derivatives and investigated them as ChEs (Supplementary Scheme S9).

Compounds **49a** (IC_50_ = 24.9 nM) and **49b** (IC_50_ = 25.9 nM) exhibited the highest activity against AChE. They were 1.9-fold more than donepezil (IC_50_ = 47.4 nM) (Supplementary Figure S6).

The presence of methyl, nitro, fluorine, and bromine groups on the benzyl moiety improved the AChE inhibitory activity compared with that of unsubstituted derivatives (**49c** (IC_50_ = 29.5 nM), **49b** (IC_50_ = 24.9 nM), **49d** (IC_50_ = 36.7 nM), **49e** (IC_50_ = 38.3 nM) vs*.* **49f** (IC_50_ = 380 nM)) (Supplementary Figure S6). Replacing the fluorine on the benzyl moiety with the nitro or bromine group somewhat diminished the inhibitory activity. Additionally, compounds having a methyl group showed a slight increase in the AChE inhibition [e.g., **49g** (IC_50_ = 37.3 nM) vs. **49h** (IC_50_ = 63.2 nM) and **49e**] (Supplementary Figure S6). Based on results, the presence of electron-donating groups on the benzyl moiety might be useful for favorite AChE inhibitory activity.

The substituents at the 4-position had the most activity against AChE. The existence of numerous groups except for the fluorine at the 4-position of the benzyl moiety significantly decreased the inhibitory activity [**49i** (IC_50_ = 29.1 nM), **49j** (IC_50_ = 119 nM), and **49k** (IC_50_ = 1,187 nM)]) (Supplementary Figure S6).

The compound **49b** (IC_50_ = 35.4 nM) displayed the highest activity against hAChE, which was as similar as donepezil (IC_50_ = 31.8 nM).

Khunnawutmanotham et al. (2018) synthesized and evaluated some coumarins conjugated with the benzylpyridinium moiety *via* an amide spacer against AChE (Supplementary Scheme S10).

**FIGURE 4:**
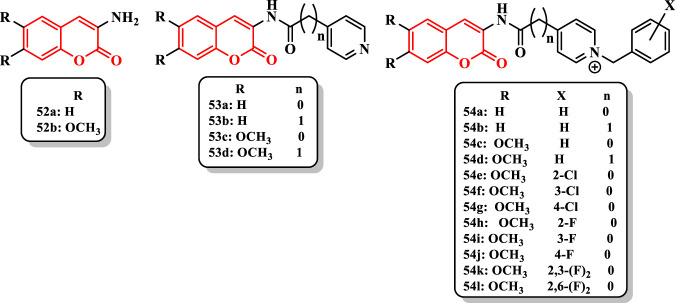
Amino-dialkoxy coumarin derivatives.

3-Aminocoumarins **52a** and **52b** showed no inhibitory activity against AChE (12% and 5%, respectively). Moreover, *N*-acyl-3-aminocoumarin derivatives such as **53a**, **53b**, **53c**, and **53d** did not increase activity (0%, 8%, 9%, and 9%, respectively). The change of **53a**–**54a** increased AChE inhibitory activity (IC_50_ = inactive to 71.88 nM). Also, the compound **54b** showed poor activity (10% inhibition). According to IC_50_ results of **54a** and **54b,** the existence of a methylene spacer between the pyridine ring and the carbonyl group diminished the inhibitory activity. The removal of the methylene group from the compound **54c** made an IC_50_ = 12.48 nM, whereas the compound **54d** showed an IC_50_ = 1,087.7 nM. Furthermore, the placement of methoxy groups at 6- and 7-positions on coumarin ring strangely improved the anti-AChE activity of compounds **54c** (IC_50_ = 12.48 nM) and **54a** (IC_50_ = 71.88 nM, and of compounds **54d** (IC_50_ = 1,087.7 nM) and **54b** (10%) (Figure 4). Compared with **54c**, the presence of chlorine at the 2-position [**54e** (IC_50_ = 6.03 nM)] led to enhanced activity while that at 3- and 4-positions [**54f** (IC_50_ = 11.47 nM) and **54g** (IC_50_ = 293.17 nM), respectively] gave similar and diminished inhibitory activities, respectively (Figure 4). Similarity, the presence of fluorine at the 2-position [**54h** (IC_50_ = 3.05 nM)] augmented the inhibitory activity, while the presence of fluorine at 3- and 4-positions diminished the activity of compounds **54i** (IC_50_ = 5.04 nM) and **54j** (IC_50_ = 5.31 nM). Difluorinated compounds were more active than monofluorinated compounds [**54k** (IC_50_ = 1.53 nM) vs. **54l** (IC_50_ = 2.43 nM)]. In addition, the compound **54k** was the most active compound in this study that the IC_50_ value was 35-fold less than donepezil (IC_50_ = 53.51 nM) and 124-fold lower than tacrine (IC_50_ = 190.37 nM) (Figure 4).

Vafadarnejad et al. (2018) synthesized different coumarin–pyridinium hybrids and investigated them as the inhibitors of ChEs (Supplementary Scheme S11).

The compound **59a** (IC_50_ = 10.14 µM compared with rivastigmine (IC_50_ = 11.07 µM)) from the 4-pyridinium series exhibited the best anti-AChE activity. The compound **59h** represented lower activity (IC_50_ = 36.82 µM). Yet, compounds **59h**, **59i** (IC_50_ = 26.25 µM), and **59j** (IC_50_ = 33.02 µM) having fluorine at 2- and 4-positions on the benzyl moiety from the 3-pyridinium series exhibited somewhat better activity. In continuance, the compound **59b** was established to exhibit the weakest anti-AChE activity (IC_50_ = 95.81 µM). The compound **59c** indicated the second potent anti-AChE activity from the 4-pyridinium series with IC_50_ = 12.15 µM, and the compound **59k** was established to be weaker (IC_50_ = 32.38 µM) (Supplementary Figure S7).

Opposite results were detected in compounds having 2-chloro and 2,3-dichlorobenzyl groups from the 3-pyridinium series (**59l** and **59m**), which displayed better inhibitory activity toward AChE (IC_50_ = 17.70 and 24.93 µM, respectively) than their analogs (**59d** and **59e**, IC_50_ = 28.66 and 54.64 µM, respectively). The compound **59f** from the 4-pyridinium series having the 2-methylbenzyl group revealed inhibitory activity with IC_50_ = 16.29 µM. The compound **59g**, in the same class, bearing 4-methylbenzyl moiety did not reveal any activity (IC_50_ > 100). Compounds **59n** and **59o** showed moderate activity (IC_50_ = 47.73 and 51.69 µM, respectively) and were weaker than their analog, the compound **59f** (IC_50_ = 16.29 µM) (Supplementary Figure S7).

Hosseini et al. (2019) reported synthesis and evaluation of a series of the 4‐oxobenzo[*d*]1,2,3‐triazin‐pyridiniums against ChEs (Supplementary Scheme S12).

Among the synthesized compounds, the compound **66a** (IC_50_ = 0.10 μM) was found as the most potent compound against AChE (Supplementary Figure S8). In the 3-pyridinium series, the compound **66b** (IC_50_ = 0.17 μM) without the substituted benzyl group revealed good anti-AChE activity. The placement of methyl or fluorine substituent at the 2-position of the benzyl group of **66b**, as in compounds **66c** (IC_50_ = 0.20 μM) and **66d** (IC_50_ = 0.29 μM), respectively, reduced the anti-AChE inhibitory activity. Furthermore, the presence of fluorine at 3- and 4-positions, bromine at the 2-position, or nitro at the 4-position on the benzyl group of the compound **66b** had good effect on the inhibitory activity of compounds **66e** (IC_50_ = 0.17 μM), **66f** (IC_50_ = 0.15 μM), **66g** (IC_50_ = 0.15 μM), and **66h** (IC_50_ = 0.16 μM) (Supplementary Figure S8). By the way, the inhibitory activities of the fluorinated analogs **66d**, **66e**, and **66f** were in the order of 4 > 3 > 2, respectively, while the inhibitory activities of the brominated analogs **66g**, **66i** (IC_50_ = 0.30 μM), and **66j** (IC_50_ = 0.84 μM) were in the order of 2 > 3 > 4, respectively (Supplementary Figure S8).

In the 4-pyridinium series, the compound **66k** showed the highest activity (IC_50_ = 1.18 μM). In this series, the derivatives **66l**, **66m**, and **66n** with hydrogen, chlorine, and nitro at the 2-position, respectively, onto the benzyl ring exhibited approximately the same anti-AChE inhibitory activity (IC_50_ = 1.34–1.37 μM). Fluorinated derivatives **66o** (IC_50_ = 1.68 μM) and **66p** (IC_50_ = 1.64 μM) were the least active derivatives in this series (Supplementary Figure S8). According to the anti-AChE activity of the 4-pyridinium series with 3-pyridinium, it was discovered that the switching of nitrogen in pyridinium ring from the 3- to the 4-position led to a significant reduction in the activity of, e.g., the compound **66k** vs. the compound **66j**.

Mollazadeh et al. (2019) described the synthesis of 2,4-dioxochroman derivatives linked to benzylpyridinium and investigated them as ChEs (Supplementary Scheme S13).

The most potent compounds had chlorine, bromine, and nitro at the 2-position on the benzyl moiety [compounds **73a** (IC_50_ = 0.89 μM), **73b** (IC_50_ = 1.10 μM), and **73c** (IC_50_ = 1.41 μM), respectively] (Supplementary Figure S9), which were less active than donepezil (IC_50_ = 0.028 μM). Furthermore, compounds **73d** (IC_50_ = 3.00 μM), **73e** (IC_50_ = 2.34 μM), **73f** (IC_50_ = 2.29 μM), and **73g** (IC_50_ = 2.54 μM) exhibited good anti-AChE activity (Supplementary Figure S9). The placement of a methyl group at the 2-position of the benzyl ring [compound **73h** (IC_50_ = 9.48 μM)] exhibited a dramatic reduction in the inhibitory activity against AChE. Shifting the methyl group from the 2- to the 4-position created **73i** (IC_50_ = 4.75 μM) that improved the inhibitory activity. The presence of fluorine at the 3-position of the benzyl ring enhanced the anti-AChE activity of the compound **73j** (IC_50_ = 2.34 μM)]. The compound **73j** having the fluorine group and the compound **73i** having the methyl group at the 4-position of the benzyl ring revealed approximately a similar anti-AChE activity (Supplementary Figure S9).

The compound **73a**, having the chlorine at the 2-position of the benzyl group revealed the highest anti-AChE activity (IC_50_ = 0.89 μM). Moving the chlorine from the 2- to the 3-position and/or inserting the second chlorine into the 3-position remarkably diminished the inhibitory activity (compound **73a** vs. compounds **73f** and **73g**). Furthermore, the presence of chlorine at the 4-position of the benzyl group [compound **73k** (IC_50_ > 100 μM)] removed the anti-AChE activity. The compound **73b** (IC_50_ = 1.10 μM) was the second most potent compound that had the bromine at the 2-position; moving the bromine from the 2- to the 3-position [compound **73l** (IC_50_ = 8.23 μM)] decreased the inhibitory activity, while attachment of the substituent at the 4-position (the compound **73l**) led to the removal of inhibitory activity (Supplementary Figure S9). According to results, the existence of an electron-withdrawing substituent with a suitable size at the 2-position of the benzyl ring can aid in making a better interaction with the AChE. The compound **73n** (IC_50_ = 12.48 μM) having CN at the 4-position on the benzyl group displayed a moderate anti-AChE activity (Supplementary Figure S9).

Shuai et al. (2019) synthesized some isothio- and isoselenochromanone derivatives having *N*-benzylpyridinium moiety (Supplementary Scheme S14).

When pyridine moiety was exchanged by piperidine, the anti-AChE activity of compounds **82a** (IC_50_ = 7,470 nM), **82b** (IC_50_ = 8,020 nM), **82c** (IC_50_ = 16,400 nM), and **82d** (IC_50_ = 15,600 nM) (Supplementary Figure S10) was dramatically reduced. The unsubstituted benzyl group compound **82e** having an isothiochromanone moiety showed the highest anti-AChE activity (IC_50_ value of 2.7 nM), which was 4.7-fold more potent than donepezil (IC_50_ = 12.7 nM). In the presence of fluorine at the 4-position on the benzyl group, the compound **82f** showed a potent inhibitory activity (IC_50_ = 5.8 nM), which was 2.2-fold more potent than donepezil.

The unsubstituted benzylpyridinium compounds **82e** and **82g** having an isothiochromanone moiety showed the highest anti-AChE activity (IC_50_ = 440 and 564 nM, respectively), which was approximately 1.5-fold more potent than donepezil (IC_50_ = 737 nM) (Supplementary Figure S10).

### Indole hybrids

Rook et al. (2010) synthesized a series of *N*2 and *N9*-bivalent β-carboline derivatives and introduced them as potent inhibitors of ChEs.

Compounds **84c** (IC_50_ = 278 nM), **84d** (IC_50_ > 10,000 nM), and **84e** (IC_50_ = 4,261 nM) with a spacer less than six carbons showed moderate activity, whereas compounds **84f** (IC_50_ = 81 nM) and **84g** (IC_50_ = 63 nM) with a spacer more than six carbons displayed stronger activities for both ChEs (Supplementary Figure S11). Substituents on the aromatic moiety (compounds **84d** and **84e**), the replacement of the tricyclic aromatic moiety with pyridinium [compounds **86** (IC_50_ = 564 nM) and **87** (IC_50_ > 10,000 nM)], and the variation of the spacer [compounds **84h** (IC_50_ = 3,141 nM) and **84i** (IC_50_ = 147 nM)] reduced the activities of the compounds (Supplementary Figure S11).

By contrast, methylation at the 2-position and introducing a permanent positive charge into the structure strongly increased the activity of the resulting compounds with spacers longer than 5 carbons [compounds **90a** (IC_50_ = 0.5 nM) and **90b** (IC_50_ = 1.2 nM)] (Supplementary Figure S11).

The *N*9-bivalent β-carbolines without a permanent positive charge (**89a-c**) generally showed very low ChEs inhibitory activities. Interestingly, partial reduction of the compound **90a** (IC_50_ = 0.5 nM for AChE and 5.7 nM for BuChE) to **91** (IC_50_ = 27 nM for AChE and 38 nM for BuChE) resulted in a moderate decrease in the ChE inhibitory activity (Supplementary Figure S11).

Yu Q. et al. (2010) synthesized the *N*-monophenylcarbamate analogs of neostigmine methyl sulfate (**92a**), pyridostigmine bromide (**92b**), and *N* (1)-methylammonium analogs of (-)-phenserine (**92d**), (-)-tolserine (**92f**), (-)-cymserine (**92h**), and (-)-phenethylcymserine (**92j**) to produce long-acting peripheral inhibitors of AChE and BuChE.

The presence of a phenylcarbamoyl in either neostigmine (**92k**) or pyridostigmine (**92l**) (for AChE and BuChE, IC_50_ = 360 nM and 900 nM, respectively), to afford analogs of **92a** and **92b** (for AChE and BuChE, IC_50_ > 30,000 nM and 550 nM, respectively), resulted in a loss of anti-ChE activity. The IC_50_ value of neostigmine (**92k**) decreased by 100- and 300-fold for AChE and BuChE (from 18.8 to 1875 nM, and from 60 to 18,000 nM), for **92a**, respectively. This similar modification in pyridostigmine (**92l**) exhibited a loss of AChE activity for **92b** but unlike **92a**, retained anti-BuChE activity (Supplementary Figure S12).

By contrast, the quaternization of (-)-physostigmine (**92m**) (for AChE and BuChE, IC_50_ = 27.9 nM and 16.0 nM, respectively) and related phenylcarbamates to provide **92n** (for AChE and BuChE, IC_50_ = 26.1 nM and 130 nM, respectively), **92d** (for AChE and BuChE, IC_50_ = 25.4 nM and 210 nM, respectively), **92f** (for AChE and BuChE, IC_50_ = 14.6 nM and 140 nM, respectively), **92h** (for AChE and BuChE, IC_50_ = 145 nM and 43 nM, respectively), and **92j** (for AChE and BuChE, IC_50_ = 300 nM and 51 nM, respectively), with charge characteristics akin to neostigmine (**92k**) and pyridostigmine (**92l**), retained or enhanced the AChE inhibitory activity. For *N* (1)-methylammonium bromides (**92n** (for AChE and BuChE, IC_50_ = 26.1 nM and 130 nM), **92d**, and **92f**) of (-)-physostigmine (**92m**), (-)-phenserine (**92c**) (for AChE and BuChE, IC_50_ = 24.0 nM and 1,560 nM, respectively), and (-)-tolserine (**92e**) (for AChE and BuChE, IC_50_ = 10.3 nM and 1950 nM, respectively), the high AChE activity of the parent compounds was kept, but the differential selectivity of **92m** and **92c** for AChE was missing resulting in the enhancement of BuChE inhibition. In the case of the BuChE-selective inhibitors, (-)-cymserine (**92g**) (for AChE and BuChE, IC_50_ = 760 nM and 51 nM, respectively), and (-)-phenethylcymserine (**92i**) (for AChE and BuChE, IC_50_ > 30,000 nM and 6.0 nM, respectively), quaternization caused a remarkable increase in the AChE inhibitory activity for **92h** and **92j** that, together with a less 10-fold against BuChE activity for **92j**, resulted in a decrease in the BuChE selectivity of these quaternary compounds (Supplementary Figure S12). Yet, the resulting AChE IC_50_ values of these quaternary (-)-physostigmine phenylcarbamates (**92n**, **92d**, **92f**, **92h**, and **92j**) were compared approvingly with those of neostigmine (**92k**) and were more potent than those of pyridostigmine (**92l**). Furthermore, the AChE and BuChE IC_50_ values were compared favorably with those of prior synthesized quaternary (-)-physostigmine phenylcarbamate iodide salts.

Khorana et al. (2012) evaluated various indoles, β-carbolines, and quinolines against AChE (Supplementary Figure S13).

For indoles with the electron-donating group such as compounds **93a** (4.26% inhibition) and **93b** (11.41% inhibition), % inhibition lower than 20% was reported, and for indoles containing electron-withdrawing groups such as compounds **93c** (36.82% inhibition) and **93d** (19.04% inhibition), % inhibition lower than 40% was obtained. It seems that the small compounds cannot be suitable for the AChE active site occupancy. Moving the substituent to the 2-position of the pyrrole ring also led to low activity, *e.g.,* 2-methylindole **93e** with %inhibition of 17.97%. The introduction of a longer substituent such as ethylamine into the pyrrole moiety and 5-methoxy into the benzene ring (**93f**, 17.19% inhibition) did not improve AChE inhibition compared with that of **93a**. However, serotonin (**93g**, 62.59% inhibition) having the hydroxyl group at the 5-position exhibited important enhancement in %inhibition compared with **93a-f**. Therefore, the more rigid structures of β-carboline derivatives were evaluated with an increase in the anti-AChE activity. Like **93h** (83.19% inhibition), they showed a good %inhibition on AChE.

The substitution in **93h** at the 7-position using methoxy (**93i**, 74.10% inhibition) did not show the anti-AChE activity, while replacement of methoxy by the hydroxyl group (**93j**, 87.07% inhibition) increased the activity. Reduction of one of the double bonds in pyridine ring (**93k**) improved the activity of about fivefold (85.52% inhibition) (Supplementary Figure S13). It showed that the flexibility of the compound in the suitable direction was essential for binding to the active site. Unlike the compound **93l** (88.61% inhibition), the reduced form of **93j** did not display the inhibitory activity. The tetrahydro-β-carboline analog (**93m**, 60.56% inhibition) exhibited the better inhibitory activity than the other less flexible β-carboline.

1-Carboxylic and 6-methoxy substituents on the tetrahydro-β-carboline ring are not suitable for desired activity as in **93n** (43.07% inhibition) and **93o** (14.29% inhibition). The 6-methoxyquinoline (**93p**) showed no inhibitory activity similar to its bioisostere **93a**. 6-Methoxy-1-methylquinolinium iodide (**93q** (87.17% inhibition) and 1-benzyl-6-methoxyquinolinium iodide (**93r** (99.68% inhibition) significantly enhanced the anti-AChE activity higher than the 6-methoxyquinoline (**93p**) with IC_50_ = 7.67 µM and 2.46 µM, respectively (Supplementary Figure S13).

In a research, Peng et al. (2012) reported various bisindole derivatives. Among them, **100b** showed enhanced activity with a Ki = 1,437.00 nM against AChE (Supplementary Figure S14.

The indole ring of **100a** was switched with a positively charged quaternary nitrogen (compounds **100g** and **100h**). These compounds did display remarkably increased activity (Ki = 197.78 and 123.73 nM, respectively), approximately 70- and 100-fold higher than **100a** (Supplementary Figure S14).

The indole ring was exchanged with acetophenone moiety (**105a**) that would play as hydrogen bond acceptors of the compound with the goal of making new interactions in the CAS additionally to the π–π stacking interactions. The activity of the compound **105a** was increased in a promising manner (Ki = 53.34 nM) (Supplementary Figure S15).

Compounds **105b-g** possessing linkers of diverse lengths, were synthesized. Compounds **105b** (Ki = 45.81 nM) and **105c** (Ki = 44.66 nM) with isoquinoline- and pyridine-substituted indole ring displayed somewhat enhanced activity, while the compound **105d** (linker contained six methylene units) was established to reveal a much higher activity (Ki = 9.41 nM). The compound **105e** having a linker one carbon longer than in the compound **105a** exhibited the maximum inhibitory activity against hAChE (Ki value of 6.47 nM), approximately with sevenfold enhanced activity than **105a** (Ki = 53.34 nM). Yet, a higher increase in the linker showed a reduction in the activity. For instance, compounds **105f** (Ki = 81.37 nM) and **105g** (Ki = 47.25 nM) having five and six methylene units revealed less activity than the compound **105e**. The compound **105e** exhibited higher activity than the compound **100a** against AChE (Supplementary Figure S15).

Mainly, the compound **105d** reduced approximately 38-fold compared with tacrine, and it displayed around 10-fold higher anti-AChE activity than anti-BuChE activity. The compound **105f** exhibited also almost the same inhibitory activity against both AChE and BuChE (Supplementary Figure S15).

**FIGURE 5:**
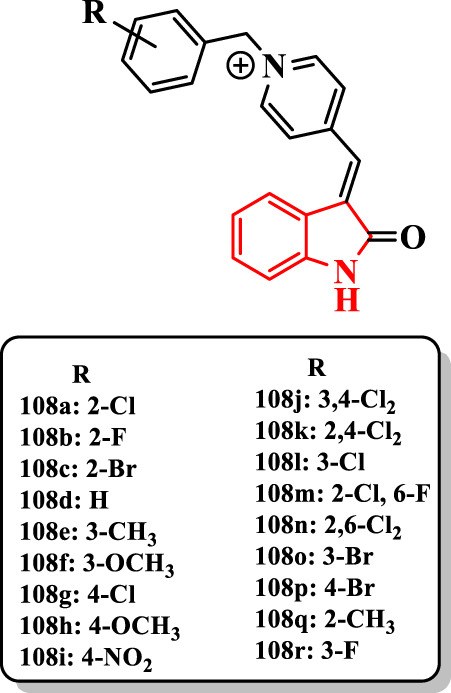
Indolinone-based compounds.

Some indolinone derivatives having benzylpyridinium moiety were evaluated as dual-binding inhibitors of AChE by Akrami et al. (2014). According to IC_50_ values, compounds **108a-d**, **108l-o**, and **108r** (IC_50_ = 0.44–12.8 nM) were more potent than donepezil (IC_50_ = 14 nM). The derivative **108a** showed the highest anti-AChE activity with an IC_50_ value of 0.44 nM (Figure 5). Furthermore, fluorine and bromine at 2-position (compounds **108b** and **108c**, respectively) with IC_50_ values 1.25 and 1.46 nM exhibited more activity than AChE. The unsubstituted compound **108d** (IC_50_ = 47.10 nM) with 2- or 3-substituted analogs established that the presence of methyl (e.g., **108e** (IC_50_ = 5.2 nM)), halide (e.g., **108a** (IC_50_ = 0.44 nM)), and methoxy (e.g., **108f** (IC_50_ = 6.6 nM)) groups at the 2- or 3-position of *N*-benzyl moiety remarkably increased the anti-AChE activity. Among them, the chlorine at the 2-position had the most effect on the AChE. Unlike, the presence of diverse substituents at the 4-position of the benzyl group reduced the AChE inhibitory activity of, *e.g.*, compounds **108g** (IC_50_ = 590 nM), **108h** (IC_50_ = 677 nM), and **108i** (IC_50_ = 744 nM). The compound **108i** bearing the nitro group at the 4-position more dramatically diminished the activity. The attachment of second chlorine at 2- and 3-positions of the 4-chlorobenzyl derivative **108g** showed a more potent activity in compounds **108j** (IC_50_ = 29.4 nM) and **108k** (IC_50_ = 257 nM). Nevertheless, compounds **108a** or **108l** (IC_50_ = 4.9 nM) bearing chlorine at 2- or 3-position on the benzyl group, respectively, showed that the presence of second halogen declined the anti-AChE activity as detected with compounds **108m** (IC_50_ = 4.1 nM), **108n** (IC_50_ = 17 nM), **108j** (IC_50_ = 29.4 nM), and **108k** (IC_50_ = 257 nM). The position of the halogen group on the benzyl moiety significantly affects the anti-AChE activity. The order of activity was as follows: 2 > 3 > 4 for, e.g., **108c** (IC_50_ = 1.46 nM), **108o** (IC_50_ = 10.3 nM), and **108p** (IC_50_ = 653.4 nM) (Figure 5).

The compound **108a** with the highest activity against AChE exhibited higher selectivity for this enzyme (SI = 3,113).

Luo et al. (2015) synthesized a series of melatonin-derived benzylpyridinium bromides (Supplementary Scheme S22).

Among all compounds, the compound **116b** (IC_50_ = 0.11 µM) exhibited the highest activity against AChE, and its potency was 10-fold less than donepezil (IC_50_ = 0.014 µM). However, **112k** revealed the most potent activity toward BuChE (IC_50_ value of 0.08 µM), which was 70-fold more than donepezil (IC_50_ = 5.6 µM). It seemed that the inhibitory activities of 4-pyridinium derivatives against hAChE [**112c** (IC_50_ = 4.2 µM) and **112d** (IC_50_ = 3.2 µM)] were more potent than those of the 3-pyridinium series [**112a** (IC_50_ = 30.5 µM) and **112b** (IC_50_ = 28.6 µM)], while the opposite trend was established on the inhibitory activity against BuChE [**112c** (IC_50_ = 2.8 µM) and **112d** (IC_50_ = 4.1 µM)) vs. **112a** (IC_50_ = 0.28 µM) and **112b** (IC_50_ = 0.34 µM)] (Supplementary Figure S11). The presence of cyanide group [**112g** (IC_50_ = 22.9 µM for AChE) (IC_50_ > 100 µM for BuChE)] at the 4-position of the benzyl group decreased strangely the inhibitory activity against ChEs (Supplementary Figure S15).

Derivatives with a methoxy group at the 5-position of the indole ring were synthesized. Compounds **112h-j** (IC_50s_ = 3.3, 3.8, and 5.1 µM, respectively, for AChE and IC_50_s = 6.8, 7.9, and 7.5 µM, respectively for BuChE) exhibited the same activity of the corresponding unsubstituted analogs (**112d-f**) (IC_50_s = 3.2, 3.9, and 3.4 µM, respectively, for AChE and IC_50_s = 4.1, 5.1, and = 4.8 µM, respectively, for BuChE) (Supplementary Figure S15). Those results indicated that the presence of methoxy at the 5-position of the indole ring exhibited a low effect on ChEs. Furthermore, the derivative **112k** (IC_50_ = 1.3 µM for AChE and IC_50_ = 0.08 µM for BuChE) having carbamate at the 4-position of the benzyl ring exhibited enhanced anti-ChE activity; particularly, the inhibitory activity of **112k** was closely 94-fold higher than that of the compound **112j** (IC_50_ = 5.1 µM for AChE and IC_50_ = 7.5 µM for BuChE) against BuChE (Supplementary Figure S11). Moreover, to study the possible activities, e.g., the effect of length of the spacer between benzylpyridinium and tryptamine on the ChE inhibitory activity, compounds **116a-f** were synthesized. It was established that both AChE and BuChE inhibitory activities of compounds **116a-c** (IC_50_s = 0.26, 0.11, and 0.21 µM, respectively, for AChE and IC_50_s = 2.3, 1.1, and 0.71 µM, respectively, for BuChE) with an additional double bond on the spacer, were improved significantly with respect to the corresponding shorter analogs (**112h-j**) (Supplementary Figure S15). Reduction of the additional double bond of compounds **116d-f** (IC_50_s = 0.53, 0.44, and 0.58 µM, respectively, for AChE and IC_50_s = 0.86, 0.72, and 0.65 µM, respectively, for BuChE) exhibited a minor reduction in anti-AChE activity and enhanced anti-BuChE activities (Supplementary Figure S15).

Salehi et al. (2019) reported a range of benzoheterocycles linked to benzylpyridinium (benzimidazole, benzoxazole, or benzothiazole) as ChE inhibitors (Supplementary Scheme S23).

Benzothiazole derivatives **120a**, **120b**, **120c**, and **120d** (Figure 6) showed a similar or more potent anti-AChE activity than donepezil (IC_50_ = 14–23 nM). The compound **120a** (IC_50_ value of 14 nM) exhibited the more potent activity against AChE. The unsubstituted compound **120a** with substituted benzyl derivatives demonstrated that the presence of diverse substituents decreased the anti-AChE activity of the compounds [e.g., compound **120e** (IC_50_ = 30 nM), compound **120f** (IC_50_ = 53 nM), and compound **120g** (IC_50_ > 300 nM)]. Based on results, the hydrophobic substituent such as chlorine at the 2-position was more suitable than others [e.g., compound **120b** (IC_50_ = 22 nM) vs. compound **120h** (IC_50_ = 159 nM)]. The shift of the methyl group from the 2- to the 3-position increased the activity of compounds **120c** (IC_50_ = 21 nM) vs. **120e** (IC_50_ = 30 nM). Compounds having halide at the 3-position of benzyl displayed that fluorine is more effective than chlorine and bromine substituents against ChE [e.g., compound **120d** (IC_50_ = 23 nM) vs. compound **120i** (IC_50_ = 61 nM) and compound **120f** (IC_50_ = 53 nM)]. In the 4-substituted congeners, the compounds **120k** (IC_50_ = 1900 nM) and **120l** (IC_50_ > 300 nM) were less potent against AChE than **120m** (IC_50_ = 36 nM). The nitro group dramatically reduced the activity of compounds **120h** and **120k**, whereas the existence of electron-withdrawing groups with small size such as fluorine on the benzyl ring showed good inhibitory activity against AChE (**120d** and **120m**). The introduction of the second chlorine into the 2- or 3-position (**120r** (IC_50_ = 78 nM) and **120n** (IC_50_ > 300 nM)] of 2-chlorobenzyl derivative **120b** showed a reduction in the anti-AChE activity, while the introduction of fluorine into the 2-position [**120o** (IC_50_ = 27 nM)] exhibited the same activity. Particularly, the comparison of the compound **120a** with the compound **120p** (IC_50_ = 147 nM) or **120q** (IC_50_ = 100 nM) indicated that the switching of sulfur with oxygen or NH did not have suitable effect on the anti-AChE activity.

Baussanne et al. (2021) reported the synthesis of *N*-alkylpyridinium-indolizine hybrids and evaluated them against ChEs (Supplementary Scheme S24).

**FIGURE 7:**
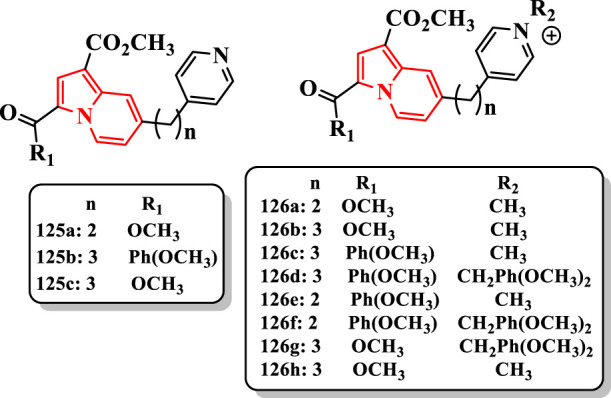
N-alkylpyridinium–indolizine hybrids.

The uncharged pyridine–indolizines (**125a-c**) were inactive against both enzymes. Compounds **126a** (IC_50_ = 5.4 µM for eeAChE and IC_50_ = 55.4 µM for eqBuChE) and its analog **126b** (IC_50_ = 7.9 µM for eeAChE and IC_50_ = 72.1 µM for eqBuChE) displayed the highest selectivity (being 10 times more active against eeAChE than against eqBuChE) (Figure 7). Donepezil as the standard drug showed IC_50_ = 2.0 µM for eeAChE and IC_50_ = 8.8 µM for eqBuChE. The **Ind-PyC3** (3 carbons between the two rings) molecules comprising the 3-*p*-methoxybenzoyl group seemed to be more active against the two enzymes than their **Ind-PyC2** (2 carbons between the two rings) analogs (**126c** (IC_50_ = 2.6 µM for eeAChE and IC_50_ = 4.8 µM for eqBuChE) and **126d** (IC_50_ > 10 µM for eeAChE and IC_50_ = 4.8 µM for eqBuChE) vs. **126e** (IC_50_ = 4.4 µM for eeAChE and IC_50_ = 17.5 µM for eqBuChE) and **126f** (inactive for both them), respectively) (Figure 7). This effect may be related to the higher flexibility of the propyl linker compared with the shorter ethyl one. The compound **126g**, analog of **126d,** exhibited a good activity against both eeAChE (IC_50_ = 2.7 μM) and eqBuChE (IC_50_ = 7.3 μM) (Figure 7). The compound **126h** was not active (IC_50_ > 100 μM) confirming its previously observed selectivity for AChE vs. BuChE.

### Phthalimide hybrids

Saeedi et al. (2016) synthesized phthalimide derivatives liked to the benzylpyridinium moiety and evaluated them against ChEs (Supplementary Scheme S25).

**FIGURE 8:**
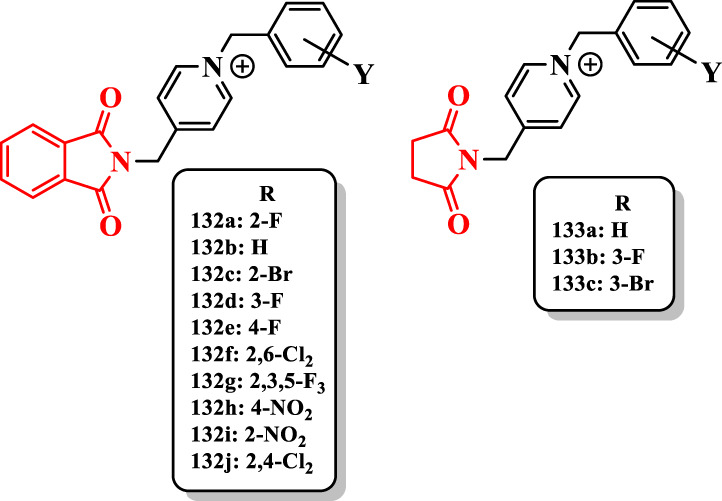
Phthalimide-based derivatives.

All compounds showed less anti-AChE activity than donepezil (IC_50_ = 0.023 µM). The compound **132a** having fluorine at the 2-position (IC_50_ = 0.77 µM) showed the highest anti-AChE activity. Its potency was fivefold more than that of unsubstituted benzyl derivative **132b** (IC_50_ = 4.29 µM). Similarly, the 2-bromobenzyl analog **132c** (IC_50_ = 2.16 µM) showed higher activity than the compound **132b**. Therefore, the presence of fluorine (**132a**) or bromine (**132c**) at the 2-position of the benzyl moiety enhanced the anti-AChE activity. However, the substitution of halogen at 3- and 4-positions had negative effect on the inhibitory activity against AChE (*e.g.*, compounds **132d** (IC_50_ = 6.14 µM) and **132e** (IC_50_ = 6.90 µM)). Among the dichlorine derivatives, analog **132f** with dichlorine at 2- and 6-positions (IC_50_ = 5.81 µM) showed better activity toward AChE (Figure 8). Reduction of activity of compounds bearing a 3- or 4-substituent may be related to the steric effects.

Derivatives bearing fluorine at the 4- or 3-position and fluorine at 2-, 3-, and 5-positions [compounds **132e**, **132d**, and **132g** (IC_50_ = 6.77 µM)] showed a similar AChE inhibitory activity. The replacement of fluorine on the benzyl group with bromine showed a decreased activity (e.g., compound **132a** vs. compound **132c**). Moreover, the presence of the nitro group at the 2- or 4-position did not increase the activity of compounds **132h** (IC_50_ = 11.23 µM) and **132i** (IC_50_ = 9.28 µM) (Figure 8).

The achieved results from the limited series of succinimide derivatives **133a** (IC_50_ = 18.3 µM), **133b** (IC_50_ = 7.19 µM), and **133c** (IC_50_ = 22.12 µM) showed that the replacement of phthalimide with succinimide decreased the anti-AChE activity. The presence of fluorine at the 3-position of the benzyl group in the succinimide series increased the anti-AChE activity (compound **132d** vs. compound **133b**) (Figure 8).

### Benzothiophene hybrids

Palin et al. (2002) synthesized some pyridinium and piperidinium salts containing 5,6-dimethoxybenzothiophene as AChE inhibitors.

The compound **134a** (IC_50_ = 0.19 µM) exhibited about a fourfold reduction in the AChE inhibition compared with **134b** (IC_50_ = 0.043 µM). The hydroxyl-containing compounds **134b** and **134a** showed fivefold to 20-fold weaker activity in the AChE inhibition than **134c** (IC_50_ = 0.008 µM) (Supplementary Figure S16).

The compound **134d** (IC_50_ = 0.09 µM) exhibited a twofold decline toward AChE **134b.** Both *exo* and *endo* compounds [**134e** (IC_50_ = 0.52 µM) and **134f** (IC_50_ = 0.75 µM)] had weak AChE inhibitory activity (0.52 and 0.75 mM, respectively) (Supplementary Figure S16). Decreasing the spacer significantly diminished the activity against AChE. All these compounds showed weaker activity than **134c** and **134b**, suggesting the propanone is the optimum linker.

The *N*-methoxyethyl and *N*-carboxymethyl derivatives (**134g** and **134h**) showed the same anti-AChE activity (IC_50_ = 0.054 and 0.053 µM, respectively). The presence of nitrofuran at the 2-position of **134i** and tetrahydropyran at that of **134j** derivatives provided the highest anti-AChE activity (IC_50_ = 0.032 and 0.02 µM, respectively). Phenoxyethyl derivative **134k** (IC_50_ = 0.39 µM) and cyanomethyl derivative **134l** (IC_50_ > 1.0 µM) showed a decrease in the inhibition of AChE (Supplementary Figure S16).

The presence of simple alkyl groups such as methyl **134m** (IC_50_ = 0.9 µM), ethyl **134n** (IC_50_ = 0.28 µM), and allyl **134o** (IC_50_ = 0.54 µM) reduced AChE inhibitory activity.

The presence of a choline (e.g., **134p** and **134r**) increased the anti-AChE activity (IC_50_ = 0.03 µM and 0.007 µM, respectively). The *in vitro* reversal potency of **134p** was also higher than the alkyl derivatives [e.g., compound **134q** (IC_50_ = 2.57 µM) and **134s** (IC_50_ = 0.11 µM)].

*N*-Benzylpyridinium **134t** exhibited high activity against AChE (IC_50_ = 0.0046 µM). The presence of fluorine at the 4-position on the benzyl ring **134u** (IC_50_ = 0.0026 µM) enhanced the AChE inhibition. However, 4-carboxyl substituent in compound **134v** (IC_50_ = 1.0 µM) diminished anti-AChE activity. Heteroaromatic derivatives **134w** and **134x** possessing thiophene and 2-nitrofuran, respectively, gave excellent AChE inhibition (IC_50_ = 0.006 and 0.007 µM, respectively) (Supplementary Figure S16).

### Benzofuranone hybrids

Nadri et al. (2013) synthesized benzofuranone-ylidene-methyl benzylpyridinium derivatives as AChE inhibitors (Supplementary Scheme S26).

**FIGURE 9:**
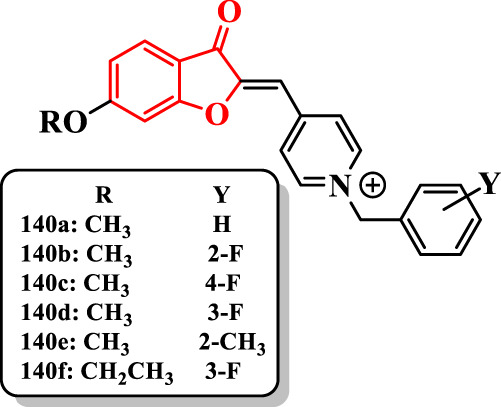
Benzofuranone-based benzylpyridinium derivatives.

The compound **140a** having the unsubstituted benzyl ring showed a significant anti-AChE activity (IC_50_ = 41 nM) compared with donepezil (IC_50_ = 28 nM). The presence of a fluorine at the 2-position [**140b** (IC_50_ = 10 nM)] or 4-position [**140c** (IC_50_ = 22 nM)] of the benzyl moiety resulted in an increase in the anti-AChE activity. The compound **140b** bearing fluorine at the 2-position of the benzyl group revealed the highest activity (IC_50_ = 10 nM) and was more potent than donepezil. Shifting the position of fluorine from the 2- to the 3-position showed a stronger decline of activity in the compound **140d** (60 nM), while the fluorine at the 4-position of the compound **140c** revealed a rather minor reduction compared with compound **140b**. The presence of the methyl group on the benzyl ring showed less activity, except the compound **140e,** which displayed moderate anti-AChE activity (IC_50_ = 68 nM) (Figure 9).

Compounds **140b** and **140c** having the methoxy group showed a higher activity than donepezil. However, increasing of the alkoxy group length revealed an unfavorable effect on AChE inhibitory activity. Moreover, it was detected that replacing the methoxy group by ethoxy and propoxy groups led to a decrease in the activity except the compound **140f** (IC_50_ = 48 nM).

The same research group (Nadri et al., 2010) reported benzofuranone derivatives linked to the pyridinium moiety. The compounds were evaluated against AChE (Supplementary Scheme S27).

The methyl on the benzyl moiety [**145a** (IC_50_ = 262 nM), **145b** (IC_50_ = 208 nM), and **145c** (IC_50_ = 514 nM)] showed weaker activity than the unsubstituted analogs [**145d** (IC_50_ = 86 nM)] (Calsolaro and Edison, 2016).

The compound **145e** (IC_50_ = 52 nM) having fluorine at the 2-position showed the most potent activity against AChE. Nevertheless, compounds having methoxy (IC_50_ = 10 nM), ethoxy (IC_50_ = 32 nM), and propoxy (IC_50_ = 50 nM) at the 6-position showed less activity than its analogs (Supplementary Figure S17).

Likewise, the fluorine at the 3- and 4-position of the benzyl moiety displayed less activity than the alkoxy at the 6-position. Moving the fluorine from the 2- to either the 3- or the 4-position reduced the activity of compounds **145f** (IC_50_ = 115 nM) and **145g** (IC_50_ = 74 nM). Therefore, methyl group showed the order of activity as follows: **145b** > **145a** > **145c**. The attachment of the methyl group to any position of benzyl moiety decreased the activity compared with unsubstituted analog (**145d**).

Baharloo et al. (2015) reported benzofuran scaffold linked to benzylpyridinium derivatives as AChE inhibitors (Supplementary Scheme S28).

The derivative **150b** with IC_50_ = 4.1 nM showed the highest activity, in which its activity was sevenfold higher than donepezil (IC_50_ = 31 nM).

The studies on the benzofuran ring showed that the presence of bromine at the 5-position reduced the activity [compounds **150a** and **150c** (IC_50_ = 16 nM) vs. **150d** (IC_50_ = 8.1 nM) and **150e** (IC_50_ = 5.8 nM)]. Furthermore, the attachment of the methoxy group to the 7-position reduced the anti-AChE activity of compounds **150f** (IC_50_ = 29.5 nM) and **150g** (IC_50_ = 10.6 nM) compared with those corresponding analogs **150c** and **150e**. Compounds **150h** (IC_50_ =9.6 nM), **150i** (IC_50_ =8.7 nM), and **150j** (IC_50_ =18.9 nM) having the fluorobenzyl group showed higher activity than compounds **150k** (IC_50_ =13.5 nM) and **150l** (IC_50_ =26.5 nM) having benzyl part, respectively. However, bromine at the 4-position of benzyl derivatives **150a** and **150f** exhibited lower activity compared with their corresponding benzyl analogs **150k** and **150l**. Furthermore, the presence of nitro at the 4-position of the benzyl moiety of compounds **150k** and **150l** resulted in higher active compounds **150d** and **150g** (Supplementary Figure S18).

Mostofi et al. (2015) described different benzofuran-based chalconoids as potential AChE inhibitors (Supplementary Scheme S29).

**FIGURE 10:**
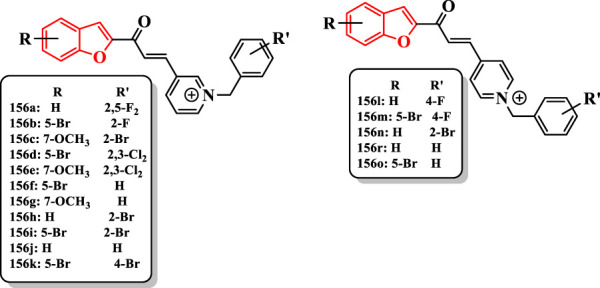
Benzofuran-based chalconoid derivatives.

Compounds were synthesized in two classes: 3-pyridinium and 4-pyridinium derivatives. The compound **156c** was potent AChE inhibitor (IC_50_ = 0.027 µM). The introduction of chlorine into the 2- and 3-positions of the pyridinium derivatives enhanced the activity of compounds**156d** (IC_50_ = 2.85 µM) and **156e** (IC_50_ = 0.985 µM) compared with that of **156f** (IC_50_ = 5.41 µM) and **156g** (IC_50_ = 3.89 µM)]; in the 4-pyridinium series, this substituent reduced the activity. The bromine at the 2-position of benzyl derivatives **156h** (IC_50_ = 0.035 µM), **156i** (IC_50_ = 2.15 µM), **156c** (IC_50_ = 0.027 µM), and **156n** (IC_50_ = 0.041 µM) showed higher activity than their benzyl analogs **156j** (IC_50_ = 0.058 µM), **156f** (IC_50_ = 5.41 µM), **156g** (IC_50_ = 3.89 µM), and **156r** (IC_50_ = 0.064 µM). Changing the position of bromine from the 2- to the 4-position of the benzyl moiety significantly diminished the activity of compound **156i** (IC_50_ = 2.15 µM) compared with that of the compound **156k** (IC_50_ = 31 µM) (Figure 10).

The compound **156c** having bromine at the 2-position of the benzyl moiety and methoxy at the 7-position of benzofuran ring revealed the highest activity (IC_50_ = 0.027 µM), which was comparable to donepezil (IC_50_ = 0.023 µM) (Figure 10).

Abedinifar et al. (2018) synthesized benzofuran-2-carboxamide-*N*-benzylpyridinium halide derivatives as ChE inhibitors (Supplementary Scheme S30).

All compounds exhibited lower AChE inhibition than donepezil (IC_50_ = 0.031 µM for AChE and 5.4 µM for BuChE), but all of them except **162a** (IC_50_ = 9.6 µM) were better BuChE inhibitors. The compound **162b** (IC_50_ = 2.1 µM) exhibited the best inhibitory activity against AChE (Supplementary Figure S19).

The introduction of the substituent into 2- and 3-positions led to higher inhibition against AChE than that of the derivative having substituent at the derivative having 4-position [e.g., compound **162b** (IC_50_ = 2.1 µM) and compound **162c** (IC_50_ = 13.8 µM) vs. compound **162d** (IC_50_ = 19.8 µM)]. The presence of strong electron-withdrawing groups at the 4-position in **162e** (IC_50_ = 40.0 µM) decreased the inhibitory activity against AChE in the 3-pyridinium series (Supplementary Figure S19).

### Bispyridinium hybrids

Musilek et al. (2011) evaluated symmetrical bispyridinium on human erythrocyte ChEs (Supplementary Scheme S31).

Among compounds, **163l**, **163m,** and **163v** (0.7–0.2 µM) showed the highest activity against hAChE. Moreover, the compound **163v** showed the same activity on hAChE like the compound **163b** (neostigmine) (IC_50_ = 0.1 µM). Moreover, the inhibitory ability of compounds **163k-m** and **163v** exceeded the frequently used commercial compound **163a** (pyridostigmine) (IC_50_ = 40 µM) (Supplementary Figure S20).

The length of the spacer was the important factor for all compounds. Among compounds **163h-l**, those having methylene units (**163k-m**) (IC_50_ = 2, 0.4, and 0.7 µM, respectively) showed the highest activity against hAChE. Compounds having shorter (**163f-h**) (IC_50_ = 505, 1,270, and 63 µM, respectively, for AChE and 9,800, 120, and 130 µM, respectively, for BuChE) or longer (**163n**) (no activity) methylene spacers were inactive against both enzymes (Supplementary Figure S20).

The length of the spacer in these compounds varied from 4 to 6 methylene units that were inadequate to interact like the compounds **163j-m** (IC_50_ = 31, 2, 0.4, and 0.7 µM, respectively, for AChE and 29, 6, 5, and 7 µM, respectively, for BuChE). The compound **163v** with a naphtylene spacer revealed the highest inhibitory activity for both enzymes (IC_50_ = 0.2 µM for AChE and 0.8 µM for BuChE) (Supplementary Figure S20). These compounds did not show selectivity for AChE over BuChE.

The same research group (Musilek et al., 2011) prepared bis-isoquinolinium ChEIs to compare their *in vitro* ability with that of standard myasthenia gravis (MG) drugs (Supplementary Scheme S32).

The compound **164h** (IC_50_ = 0.005 µM) having an aliphatic spacer showed the most potent activity against AChE, but the selectivity of the compound was poor. The most potent compounds **164h-j** (IC_50_ = 0.005, 0.04, and 0.05 µM, respectively, for AChE and 0.4, 0.6, and 1.6 µM, respectively, for BuChE) displayed only poor selectivity of AChE over BuChE (Supplementary Figure S21).

Among compounds **164d-i**, those having methylene units [**164f-k** (IC_50_ = 0.3, 0.5, 0.005, 0.04, 0.05, and 0.1 µM, respectively)] revealed the most potent inhibitory activity against hAChE. Compounds having shorter spacers [**164a-d** (IC_50_ = 654, 0, 446, and 36 µM, respectively, for AChE and 1,400, 0, 2,600, and 40 µM, respectively, for BuChE)] were found to be ineffective toward both enzymes.

The compound **164t** (IC_50_ = 0.3 µM for AChE and 4 µM for BuChE) having a naphthalenyl spacer showed an improvement in the inhibitory activity against both enzymes. The linker length of the compound **164t** was the same as compound **164g** (7 C-C bonds) and therefore exhibited the same binding to the AChE or BuChE. These compounds depicted no selectivity for AChE over BuChE.

This group (Musilek et al., 2011) also reported a series of **SAD-128** analogs as ChEIs (Supplementary Scheme S33).

The commercial oximes [pralidoxime (IC_50_ = 878 µM) and obidoxime (IC_50_ = 577 µM)] showed a weak inhibition toward hAChE, while the selective standards (**BW284c51** (IC_50_ = 0.03 µM) and ethopropazine (IC_50_ = 1,020 µM)) were favorite inhibitors of hAChE (Supplementary Figure S22).

Some synthesized compounds [**165g** (IC_50_ = 0.016 µM), **165h** (IC_50_ = 0.005 µM), **165j** (IC_50_ = 0.012 µM), **165k** (IC_50_ = 0.026 µM), **165l** (IC_50_ = 0.007 µM), and **165t** (IC_50_ = 0.024 µM)] exhibited good inhibitory activity against hAChE. Compounds **165h**, **165j**, and **165l** having an aliphatic spacer revealed the highest activity against AChE, and the compound **165t** showed the highest activity with diverse spacers.

According to results, compounds with short spacer C1-C5 (**165a-e**), that are aliphatic with heteroatom (**165m** and **165n**), and with double bonded linkers (**165o** and **165p**) or linkers bearing xylene moiety (**165q-s**) were found to be effective as hAChE inhibitors. By contrast, compounds bearing longer aliphatic C6-C12 (**165f-l**) and naphtylene linkers (**165t**) showed the highest activity against eeAChE. The even spacers C8-C10-C12 (**165h**, **165j**, and **165l**) displayed higher activity than odd spacers (**165g**, **165i**, and **165k**). Moreover, the naphthalenyl-connected compound **165t** revealed slightly lower activity than aliphatic-connected compounds **165g,** d **165h**, **165j**, and **165l**.

Komloova et al. (2013) investigated the isoquinolinium–pyridinium and quinolinium–pyridinium bisquaternary compounds **168a-k** *in vitro* against ChEs (Supplementary Scheme S34).

There are two classes of the bisquaternary derivatives: Quinolinium-pyridinium and isoquinolinium-pyridinium groups (Supplementary Figure S23). Commonly, isoquinolinium-pyridinium compounds exhibited slightly less activity toward AChE than quinolinium-pyridinium compounds. Each compound with odd number of methylene groups in the spacer had a slightly lower activity than those compartments having the even number.

Compounds with 10 methylene groups (**168c**, IC_50_ = 0.005 µM) and 12 methylene groups (**168d**, IC_50_ = 0.0047 µM) in the spacer demonstrated the most potent activity, edrophonium (IC_50_ = 5.17 µM) and **BW284C51** (IC_50_ = 0.03 µM) (Supplementary Figure S23). Furthermore, both compounds revealed the high selectivity for hAChE.

### Pyridinium hybrids

Parlar et al. (2016) synthesized some hydrazone derivatives possessing pyridinium moiety and investigated their activity against ChEs (Supplementary Scheme S35).

Compounds **170d** and **170e** having benzofuran ring showed the highest activity against eeAChE. Furthermore, **170d** and **170e** (IC_50_ = 0.32 and 0.23 μM, respectively) revealed higher activity than galantamine (IC_50_ = 0.43 μM) (Supplementary Figure S24).

Considering results from hAChE inhibitory activity all compounds made the same inhibitory activity against eeAChE. Moreover, compounds **170d** (IC_50_ = 0.62 μM) and **170e** (IC_50_ = 0.24 μM) showed the highest activity toward hAChE. The AChE inhibitory activity improved when the length of linker between pyridinium nitrogen and the benzyl ring was increased from two to three methylene units (Supplementary Figure S24). Replacing the methylene group by ether in propyl chain resulted in a strange reduction of AChE inhibitory activity.

Compounds **170a** (IC_50_ = 0.84 µM), **170b** (IC_50_ = 0.74 µM), **170d** (IC_50_ = 0.32 µM), and **170e** (IC_50_ = 0.23 µM) showed the most potent activity against AChE and exhibited the most selectivity over BuChE.

**FIGURE 11:**
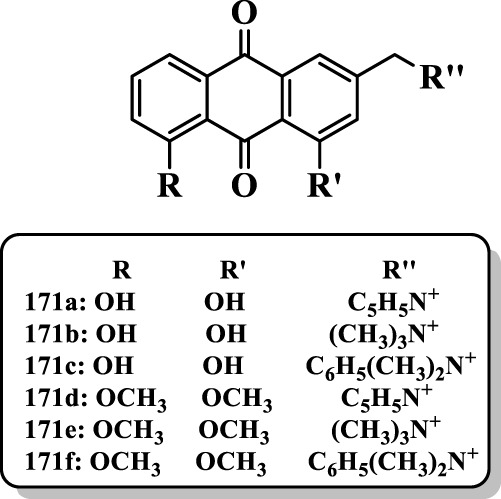
Aloe emodin derivatives.

Shi et al. (2013) synthesized various aloe emodin compounds and investigated them against AChE. Most of the compounds displayed significant AChE inhibitory activities toward AChE. The results showed that the phenolic hydroxyl groups of the aloe-emodin played a vital role in the AChE inhibitory activity. The aloe-emodin derivatives containing quaternary ammonium fragment **171a-f** (IC_50_ = 0.09, 3.76, 25.38, 0, 0.54, and 13.56 µM, respectively) showed potent AChE inhibitory activity. The compound **171a** possessed the best AChE inhibitory activity with IC_50_ value of 0.09 µM, which was higher than the positive control tacrine (IC_50_ = 0.26 µM) (Figure 11).

Lan et al. (2017a) reported a series of cinnamic acid derivatives to be multifunctional cholinesterase inhibitors against AD by linking to the *N*-benzylpyridinium part and diverse substituted cinnamic acids (Supplementary Scheme S36).

**FIGURE 12:**
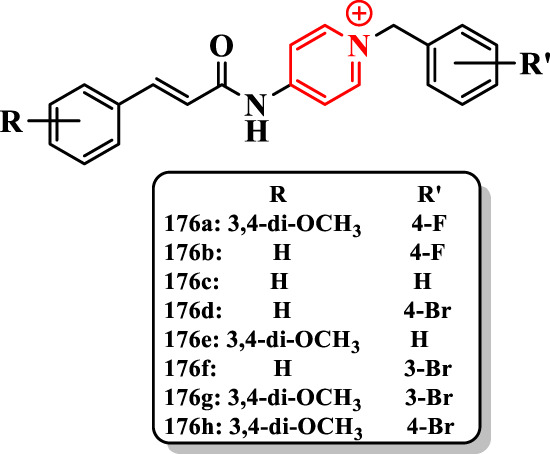
Cinnamic acid derivatives.

The compound **176a** (IC_50_ = 12.1 nM) exhibited the highest activity against AChE, and it was 3.3-fold more potent than donepezil (IC_50_ = 40.2 nM). Furthermore, the compound **176a** revealed the high selectivity for AChE over BuChE. The compound **176h** showed the most potent activity against BuChE (IC_50_ = 1.9 µM), which was 2.3-fold higher than donepezil (IC_50_ = 4.5 µM). Yet, cinnamic acid displayed significantly lower anti-AChE activity (IC_50_ > 100 µM), so it seems that the *N*-benzylpyridinium part is inescapably essential for higher activity (Figure 12).

Based on the IC_50_ results, compounds **176a-n** having methoxy at 3- and 4-positions of the benzylpyridinium part showed excellent anti-ChE activity. For instance, compound **176a** (IC_50_ = 12.1 nM for AChE and 2.6 µM for BuChE) was more potent than compound **176b** (IC_50_ = 90.8 nM for AChE and 10.0 µM for BuChE) against ChEs. The presence of fluorine, methyl, and bromine at 3- and 4-positions of the benzyl moiety declined the anti-AChE activity of compound **176c** (IC_50_ = 54.1 nM for AChE), which was higher than that of the compound **176d** (IC_50_ = 1,450.5 nM for AChE), while compounds with diverse groups on the benzyl moiety depicted an improved anti-AChE activity (compound **176a** (IC_50_ = 12.1 nM for AChE) having fluorine at the 4-position of the benzyl moiety displayed higher AChE inhibition (11-fold) than compound **176e)**. Also, compounds **176f** (IC_50_ = 2.5 µM), **176d** (IC_50_ = 2.1 µM), **176a** (IC_50_ = 2.5 µM) and **176g** (IC_50_ = 1.9 µM) bearing bromine substituent revealed potent inhibitory activity.

The compound **176a** (IC_50_ = 8.6 nM for hAChE) showed the most potent activity, which was 4-fold higher than donepezil (IC_50_ = 33.5 nM) (Figure 12).

Ghotbi et al. (2020) synthesized compounds containing thiazole and pyridinium moieties (Supplementary Scheme S37).

The derivative **183a** (IC_50_ = 0.40 μM) having fluorine at the 2-position showed the highest anti-AChE activity, and compound **183b** possessing bromine at the 2-position (IC_50_ = 0.69 μM) was the second potent inhibitor. In both compounds, fluorine and bromine were at the 2-position of the benzyl ring. Changing these substituents in **183a** and **183b** to 3 (**183c** and **183d**)- and 4 [**183e** (IC_50_ = 6.48 μM and **183f** (IC_50_ = 30.49 μM)]-positions revealed a slight reduction of inhibitory activity. According to results, the 2- and 3-substituted derivatives provided stronger inhibition against AChE than the 4-substituted derivatives (the order of substitution position is 2 > 3 > 4). For derivatives **183g** (IC_50_ = 40.80 μM) as well as **183e**, **183h** (IC_50_ = 21.49 μM), and **183f** (IC_50_ = 30.49 μM), containing nitro or halide group at the 4-position, the electron-withdrawing group decreased the activity (Supplementary Figure S25).

The presence of nitro at the 4-position of the compound showed lower activity (IC_50_ = 54.58 μM) than the halogenated derivatives, and among the diverse halogen groups, fluorine revealed the most potent anti-AChE activity to the compound **183l** (IC_50_ = 1.95 μM), while the compound **183m** unsubstituted in the 3-pyridinium series exhibited the most potent anti-AChE activity (IC_50_ = 1.64 μM) (Supplementary Figure S25). Commonly, in the 3-pyridinium series, similar to 4-substituted compounds in 4-pyridinium derivatives, the presence of a substituent led to the decreased activity.

Abdullaha et al. (2020) described the synthesis of pyridinium benzamides and screened for the inhibition of ChEs (Supplementary Schemes S37, 38).

Donepezil showed IC_50_ = 0.049 µM for the inhibition of AChE and IC_50_ = 5.52 µM for the inhibition of BuChE.

As a general trend, the 2-substituted derivatives (e.g., **187x** (88.59% for AChE), **187z** (91.67% for AChE), **191ac** (90.68% for AChE), and **191af** (49.27% for AChE)) were superior to other substitutions. The comparison of this series of compounds with the parent lead **187w** indicated that only derivatives bearing substituent at the 2-position of benzyl ring displayed a similar AChE inhibitory activity to that of **187w** (Supplementary Figure S26). The ethylene linker [**191ah** (78.93% for AChE and 53.25% for BuChE)] was significantly superior to methylene linker [**191 ag** (48.33% for AChE and 2.41% for BuChE)]. However, several compounds [**191ah**, **191aj** (78.69% for AChE and 54.17% for BuChE), and **191ao** (46.58% for AChE and 47.75% for BuChE)] displayed superior inhibition of BuChE to benzamide **187w** (Supplementary Figure S26). This series provided critical information that placing a small -CH_2_CH_2_- linker between naphthylamide and pyridine ring imparts a significant positive impact on the potency. This additional spacer might be helping the compound to occupy the active site gorge perfectly, and helping it to interact with all key residues of both the active sites.

Next, when the pyridinium moiety of the first stage lead compound **187w** was replaced with piperidine (compound **193a**), the ChE inhibitory activity was completely lost (7.70% for AChE and 1.77% for BuChE). However, the introduction of an ethylene linker between piperidine ring and naphthylamide moiety [compound **193b** (80.02% for AChE and 60.98% for BuChE)] resulted in the gain of activity against both ChEs (Supplementary Figure S26). In fact, the compound **193b**, though it does not bear quaternary nitrogen, still displayed the same level of AChE inhibition, and superior BuChE inhibition compared with **187w**. The replacement of naphthyl (compound **187w**) with phenoxy-phenyl, biphenyl, benzoyloxy-phenyl, and phenoxy-benzyl (**191aq-at**) resulted in a loss of activity, indicating that naphthalene ring is essential for dual cholinesterase inhibition.

Zarei et al. (2021) reported the synthesis of benzyl-oxoquinazolin-pyridinium derivatives assessed as ChEs inhibitors (Supplementary Scheme S40).

**FIGURE 13:**
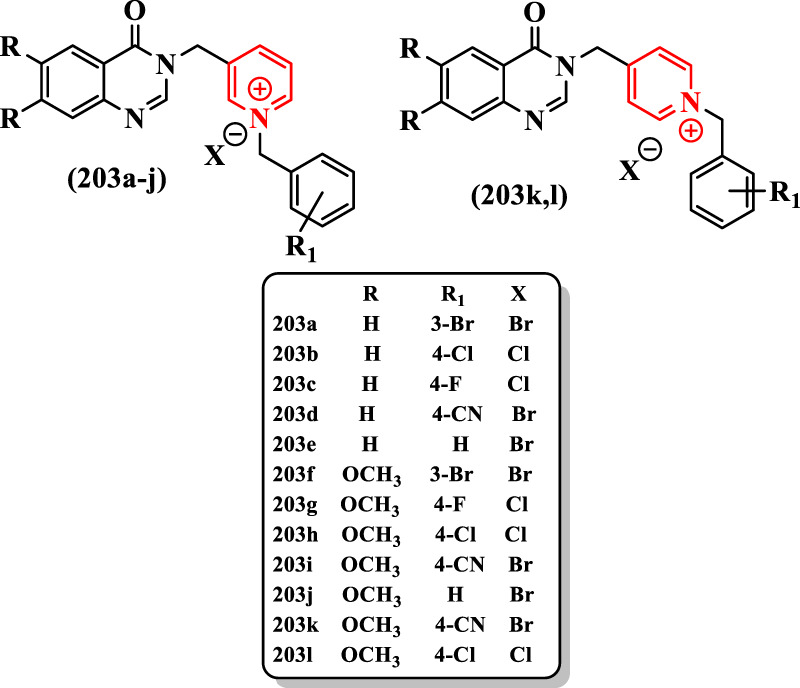
Moieties of compounds oxoquinazoline and pyridinium.

**203a-l** could be categorized into two group: 1) having methoxy group substituted on oxoquinazoline ring and 2) without methoxy group on oxoquinazoline ring. In the first group of tested compounds **203a-e**, **203a** having bromine at the 3-position of benzyl group showed the strongest AChE inhibitory effect (IC_50_ = 5.90 μM) (Figure 13). Donepezil showed IC_50_ = 0.079 µM for the inhibition of AChE and IC_50_ = 5.19 µM for the inhibition of BuChE.

Supplanting the chlorine by the fluorine in **203c** (IC_50_ > 100 µM) or CN in **203d** (IC_50_ > 100 µM) resulted in derivatives with very low activities. Although the removal of this group from the benzyl ring led to an increase in the AChE inhibition for the compound **203e** (IC_50_ = 41.21 μM), the compound **203a** remained the most potent AChE inhibitor in this group of compounds.

In the second group containing methoxy substitution on oxoquinazoline ring, the best compound was **203h** with a chlorine group at the 4-position, which showed a promising potency as an AChE inhibitor (IC_50_ = 1.11 μM). However, **203h** had no inhibitory activity against BuChE, which indicated that this compound is a good selective AChE inhibitor. The replacement of chlorine with hydrogen, fluorine, and CN at the 4-position caused a depletion of inhibitory activity for AChE in compounds **203j**, **203g**, and **203i** with IC_50_ = 10.08 μM, IC_50_ = 21.92 μM, and IC_50_ > 100 μM, respectively. The **203f** with bromine at the 3-position of the benzyl ring revealed an intermediate potency among other compounds in this group (IC_50_ = 6.77 μM).

Two compounds with 4-(methyl)pyridine moiety (**203k** and **203l**) showed lower inhibitory activities both for AChE and for BuChE than other compounds containing the 3-(methyl)pyridine group (IC_50_ > 100 µM for both enzyme). According to the observed results, on the one hand, the first group had a higher activity for BuChE inhibition. On the other hand, the second group with the substituted methoxy groups had better results for AChE inhibition. It is worthwhile to note that compounds **203s** in the series of 3-methylpyridines showed superior activity to 4-methylpyridine derivatives and the electron-withdrawing CN group decreased the inhibitory activity. Finally, the compound **203a** with IC_50_ = 5.90 μM for AChE and IC_50_ = 6.76 μM for BuChE was the most potent dual inhibitor and the compound **203h** with IC_50_ = 1.11 μM for AChE was the strongest derivative among tested compounds against AChE (Figure 13).

Hassanzadeh et al. (2021) reported synthesis of isoindoline-1,3-dione-*N*-benzylpyridinium hybrids and evaluated them against AChE (Supplementary Scheme S41).

**FIGURE 14:**
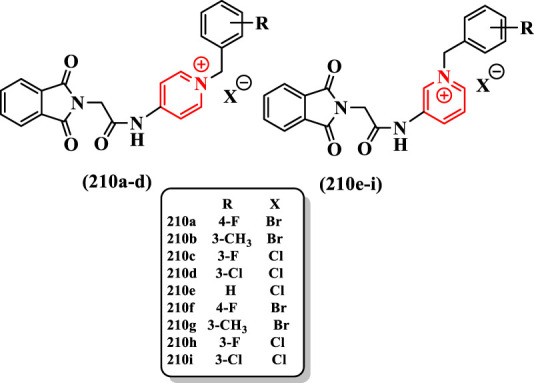
Isoindoline-1,3-dione–N-benzylpyridinium hybrid compounds.

The best anti-AChE activity was obtained by compounds **210a** and **210f** (IC_50_ = 2.1 μM possessing fluorine at the 4-position of benzylpyridinium moiety, compared with rivastigmine (IC_50_ = 11.07 μM). The shift of the fluorine group from the 4- to the 3-position in **210c** and **210h** led to a reduction in the inhibitory activity (IC_50s_ = 2.7 and 2.9 μM, respectively). The switching from the fluorine to the chlorine group in compounds **210d** (IC_50_ = 7.4 μM) and **210i** (IC_50_ = 6.7 μM) led to a more than twofold reduction in the inhibitory activity than in **210c** and **210h** so that chlorine-substituted derivatives showed the weakest inhibitory activity among compounds. The 3-methyl-substituted compounds **210b** (IC_50_ = 5.4 μM) and **210g** (IC_50_ = 4.8 μM) showed improved AChE inhibitory activities compared with chlorine-substituted compounds **210d** and **210i** (Figure 14).

Molecular docking results, kinetic analysis, and BuChE activity of reported compounds are given in the Supplementary Data section.

## Conclusion

According to the reported results in the literature, both anti-AChE and anti-BuChE activities showed almost similar SAR. AChE inhibitory activity of reported compounds was sensitive to the electronic and steric properties of substituents at different positions of the benzyl moiety of pyridinium salts. Thus, it could be concluded that irrespective of the electronic properties and the size of substituents on the benzyl group are important in inducing desired inhibitory activity. According to results, all compounds exhibited high selectivity for AChE.

1 Benzylpiperidine moiety of donepezil seems to be important for optimal AChE inhibitory activity since various studies have shown that donepezil binds to the CAS and PAS of the enzyme.
2 Dual site binding donepezil-based scaffolds have been found to elicit other vital pharmacological activities such as prevention of amyloid aggregation, which is crucial for the management of AD. Furthermore, various hybrids have also displayed MAO, BuChE inhibitory activity, metal chelating, and ROS scavenging ability.
3 Based on results, the inhibitory activity of compounds toward ChEs was sensitive to the length of the spacers between different moieties.
4 The type of functional group in the spacer, linking benzylpyridine group to other moieties, is an imperative parameter in the inhibitory activity. Generally, the presence of the amide group has depicted a positive result.
5 It was found that the type and position of substituents on the benzyl group is very important in AChE inhibitory activity. The presence of substituents at 2- or 4- or 2- and 3- positions of the aryl ring affected the activity. Substitution at 2- or 4- or 2- and 3-positions by halides was found to increase the selectivity and inhibitory activity toward AChE. The introduction of NO_2_ into these positions decreased activity. In addition, substitution at the 3-position of aryl ring seems to be unfavorable for activity. Also, the presence of substitution on benzylpyridine moiety increased anti-ChE activity in comparison with nonsubstituted compounds. The position and type of substituents on the benzyl part could change the steric and electronic properties of the aryl ring. Then, the affinity of the compounds could be improved by altering the substituent on the benzyl moiety.
6 Most derivatives showed stronger inhibitory activity than donepezil. It seems that the presence of substituents on the aryl ring and spacers in the corresponding compounds, which are not present in the donepezil structure, play important roles in the anti-ChE activity.
7 Replacing phthalimide by succinimide reduced the anti-AChE activity. It was perceived that the aromatic ring linked to the succinimide moiety played a vital role in the inhibitory activity of compounds.
8 Generally, 4-pyridinium salts series exhibited the highest anti-AChE activity and the high selectivity for AChE over BuChE compared with 3-pyrimidinum series.
9 The presence of a positive charge on the pyridinium ring is essential for inducing the inhibitory activity. Based on the results, uncharged structures showed lower ChE inhibitory activity.
10 Docking and kinetic studies exhibited that all derivatives attached to the CAS and PAS of the enzyme active site.
11 The geometric orientation of the benzylpyridinium moiety is an imperative parameter in the inhibitory activity.

It is concluded that benzylpyridinium moiety that linked to other moieties and functional groups through a spacer with appropriate length demonstrated potent AChE inhibitory activity as donepezil analogs.

And based on studies, the benzylpyridinium moiety of inhibitors interacts with the CAS and other moieties interact with the PAS of the AChE.

## References

[B1] Abdullaha M. Nuthakki V. K. Bharate S. B. (2020). Discovery of methoxy-naphthyl linked N-(1-benzylpiperidine) benzamide as a blood-brain permeable dual inhibitor of acetylcholinesterase and butyrylcholinesterase. Eur. J. Med. Chem. 207, 112761. 10.1016/j.ejmech.2020.112761 32942070

[B2] Abedinifar F. Farnia S. M. F. Mahdavi M. Nadri H. Moradi A. Ghasemi J. B. (2018). Synthesis and cholinesterase inhibitory activity of new 2-benzofuran carboxamide-benzylpyridinum salts. Bioorg. Chem. 80, 180–188. 10.1016/j.bioorg.2018.06.006 29929079

[B3] Agatonovic-Kustrin S. Kettle C. Morton D. W. (2018). A molecular approach in drug development for Alzheimer’s disease. Biomed. Pharmacother. 106, 553–565. 10.1016/j.biopha.2018.06.147 29990843

[B4] Akrami H. Mirjalili B. F. Khoobi M. Nadri H. Moradi A. Sakhteman A. (2014). Indolinone-based acetylcholinesterase inhibitors: Synthesis, biologicalactivity and molecular modeling. Eur. J. Med. Chem. 84, 375–381. 10.1016/j.ejmech.2014.01.017 25036795

[B5] Alipour M. Khoobi M. Foroumadi A. Nadri H. Moradi A. Sakhteman A. (2012). Novel coumarin derivatives bearing *N*-benzyl pyridinium moiety: potent and dual binding site acetylcholinesterase inhibitors. Bioorg. Med. Chem. 20, 7214–7222. 10.1016/j.bmc.2012.08.052 23140986

[B6] Alipour M. Khoobi M. Nadri H. Sakhteman A. Moradi A. Ghandi M. (2013). Synthesis of some new 3-coumaranone and coumarin derivatives as dual inhibitors of acetyl- and butyrylcholinesterase. Arch. Pharm. Weinh. 346, 577–587. 10.1002/ardp.201300080 23852709

[B7] Arab S. Sadat-Ebrahimi S. E. Mohammadi-Khanaposhtani M. Moradi A. Nadri H. Mahdavi M. (2015). Synthesis and evaluation of chroman-4-one linked to N-benzyl pyridinium derivatives as new acetylcholinesterase inhibitors. Arch. Pharm. Weinh. 348, 643–649. 10.1002/ardp.201500149 26192069

[B8] Baharloo F. Moslemin M. H. Nadri H. Asadipour A. Mahdavi M. Emami S. (2015). Benzofuran-derived benzylpyridinium bromides as potentacetylcholinesterase inhibitors. Eur. J. Med. Chem. 93, 196–201. 10.1016/j.ejmech.2015.02.009 25681712

[B9] Bartus R. T. (2000). On neurodegenerative diseases, models, and treatment strategies: Lessons learned and lessons forgotten a generation following the cholinergic hypothesis. Exp. Neurol. 163, 495–529. 10.1006/exnr.2000.7397 10833325

[B10] Baussanne I. Firstova O. Dediu A. B. Larosa C. Furdui B. Ghinea I. O. (2021). Interest of novel *N*-alkylpyridinium-indolizine hybrids in the field of Alzheimer’s disease: Synthesis, characterization and evaluation of antioxidant activity, cholinesterase inhibition, and amyloid fibrillation interference. Bioorg. Chem. 116, 105390. 10.1016/j.bioorg.2021.105390 34670332

[B11] Bekris L. M. Yu C. E. Bird T. D. Tsuang D. W. (2010). Review article: genetics of alzheimer disease. J. Geriatr. Psychiatry Neurol. 23, 213–227. 10.1177/0891988710383571 21045163 PMC3044597

[B12] Brus B. Kosak U. Turk S. Pislar A. Coquelle N. Kos J. (2014). Discovery, biological evaluation, and crystal structure of a novel nanomolar selective butyrylcholinesterase inhibitor. J. Med. Chem. 57, 8167–8179. 10.1021/jm501195e 25226236

[B13] Cacabelos R. (2007). Donepezil in Alzheimer’s disease: From conventional trials to pharmacogenetics. Neuropsychiatr. Dis. Treat. 3, 303–333. 19300564 PMC2654795

[B14] Calsolaro V. Edison P. (2016). Neuroinflammation in Alzheimer's disease: Current evidence and future directions. Alzheimer's. Dement. 12, 719–732. 10.1016/j.jalz.2016.02.010 27179961

[B15] Costanzo P. Cariati L. Desiderio D. Sgammato R. Lamberti A. Arcone R. (2016). Design, synthesis, and evaluation of donepezil-like compounds as AChE and BACE-1 inhibitors. ACS Med. Chem. Lett. 7, 470–475. 10.1021/acsmedchemlett.5b00483 27190595 PMC4867475

[B16] Dubey S. K. Kharbanda M. Dubey S. K. Mathela C. S. (2010). A new commercially viable synthetic route for donepezil hydrochloride: anti-Alzheimer’s drug. Chem. Pharm. Bull. 58 (9), 1157–1160. 10.1248/cpb.58.1157 20823593

[B17] Garcia-Ayllon M. S. Small D. H. Avila J. Saez-Valero J. (2011). Revisiting the role of acetylcholinesterase in Alzheimer’s disease: Cross-talk with P-tau and β-amyloid. Front. Mol. Neurosci. 4, 22. 10.3389/fnmol.2011.00022 21949503 PMC3171929

[B18] Ghotbi G. Mahdavi M. Najaf Z. Moghadam F. H. Hamzeh-Mivehroud M. Davaran S. (2020). Design, synthesis, biological evaluation, and docking study of novel dualacting thiazole-pyridiniums inhibiting acetylcholinesterase and β-amyloid aggregation for Alzheimer’s disease. Bioorg. Chem. 103, 104186. 10.1016/j.bioorg.2020.104186 32890993

[B19] Girek M. Szymanski P. (2019). Tacrine hybrids as multi-target-directed ligands in Alzheimer’s disease: Influence of chemical structures on biological activities. Chem. Pap. 73, 269–289. 10.1007/s11696-018-0590-8

[B20] Goyal D. D. Shuaib S. Mann S. Goyal B. (2017). Rationally designed peptides and peptidomimetics as inhibitors of amyloid-β (Aβ) aggregation: potential therapeutics of Alzheimer’s disease. ACS Comb. Sci. 19, 55–80. 10.1021/acscombsci.6b00116 28045249

[B21] Hassanzadeh M. Hassanzadeh F. khodarahmi G. A. Rostami M. Azimi F. Nadri H. (2021). Design, synthesis, and bio-evaluation of new isoindoline-1, 3-dione derivatives as possible inhibitors of acetylcholinesterase. Res. Pharm. Sci. 16, 482–492. 10.4103/1735-5362.323915 34522196 PMC8407153

[B22] Hosseini F. Ramazani A. Mohammadi-Khanaposhtani M. Tehrani M. B. Nadri H. Larijani B. (2019). Design, synthesis, and biological evaluation of novel 4-oxobenzo[*d*]1, 2, 3-triazin-benzylpyridinum derivatives as potent anti-Alzheimer agents. Bioorg. Med. Chem. 27, 2914–2922. 10.1016/j.bmc.2019.05.023 31128990

[B23] Jiang X. Y. Chen T. K. Zhou J. T. He S. Y. Yang H. Y. Chen Y. (2018). Dual GSK-3β/AChE inhibitors as a new strategy for multitargeting anti-Alzheimer’s disease drug discovery. ACS Med. Chem. Lett. 9, 171–176. 10.1021/acsmedchemlett.7b00463 29541355 PMC5846044

[B24] Khoobi M. Alipour M. Sakhteman A. Nadri H. Moradi A. Ghandi M. (2013). Design, synthesis, biological evaluation and docking study of 5-oxo-4, 5-dihydropyrano[3, 2-c]chromene derivatives as acetylcholinesterase and butyrylcholinesterase inhibitors. Eur. J. Med. Chem. 68, 260–269. 10.1016/j.ejmech.2013.07.038 23988409

[B25] Khorana N. Changwichit K. Ingkaninan K. Utsintong M. (2012). Prospective acetylcholinesterase inhibitory activity of indole and its analogs. Bioorg. Med. Chem. Lett. 22, 2885–2888. 10.1016/j.bmcl.2012.02.057 22425563

[B26] Khunnawutmanotham N. Chimnoi N. Saparpakorn P. Techasakul S. (2016). Synthesis and anti-acetylcholinesterase activity of scopoletin derivatives. Bioorg. Chem. 65, 137–145. 10.1016/j.bioorg.2015.12.002 26943478

[B27] Khunnawutmanotham N. Laongthipparos C. Saparpakorn P. Chimnoi N. Techasakul S. (2018). Synthesis of 3-aminocoumarin-N-benzylpyridinium conjugates with nanomolar inhibitory activity against acetylcholinesterase. Beilstein J. Org. Chem. 14, 2545–2552. 10.3762/bjoc.14.231 30410615 PMC6204823

[B28] Knowles J. (2006). Donepezil in Alzheimer’s disease: An evidence-based review of its impact on clinical and economic outcomes. Core Evid. 1, 195–219. 22500154 PMC3321665

[B29] Komloova M. Horova A. Hrabinova M. Jun D. Dolezal M. Vinsova J. (2013). Preparation, *in vitro* evaluation and molecular modelling of pyridinium-quinolinium/isoquinolinium non-symmetrical bisquaternary cholinesterase inhibitors. Bioorg. Med. Chem. Lett. 23, 6663–6666. 10.1016/j.bmcl.2013.10.043 24220173

[B30] Kryger G. Silman I. Sussman J. L. (1999). Structure of acetylcholinesterase complexed with E2020 (Aricept): Implications for the design of new anti-alzheimer drugs. Structure 15, 297–307. 10.1016/s0969-2126(99)80040-9 10368299

[B31] Lan J. S. Hou J. W. Liu Y. Ding Y. Zhang Y. Lia L. (2017). Design, synthesis and evaluation of novel cinnamic acid derivatives bearing *N*-benzyl pyridinium moiety as multifunctional cholinesterase inhibitors for Alzheimer’s disease. J. Enzyme Inhib. Med. Chem. 32, 776–788. 10.1080/14756366.2016.1256883 28585866 PMC6009898

[B32] Lan J. S. Zhang T. Liu Y. Yang J. Xie S. S. Liu J. (2017). Design, synthesis and biological activity of novel donepezil derivatives bearing N-benzyl pyridinium moiety as potent and dual binding site acetylcholinesterase inhibitors. Eur. J. Med. Chem. 133, 184–196. 10.1016/j.ejmech.2017.02.045 28388521

[B33] Lan J. S. Ding Y. Liu Y. Kang P. Hou J. W. Zhang X. Y. (2017). Design, synthesis and biological evaluation of novel coumarin-Nbenzyl pyridinium hybrids as multi-target agents for the treatment of Alzheimer's disease. Eur. J. Med. Chem. 139, 48–59. 10.1016/j.ejmech.2017.07.055 28797883

[B34] Lavado L. Zhang M. H. Patel K. Khan S. Patel U. K. (2019). Biometals as potential predictors of the neurodegenerative decline in Alzheimer’s disease. Cureus 11, e5573. 10.7759/cureus.5573 31695992 PMC6820671

[B35] Lee J. K. Kim N. J. (2017). Recent advances in the inhibition of p38 MAPK as a potential strategy for the treatment of Alzheimer’s disease. Molecules 22, 1287. 10.3390/molecules22081287 PMC6152076 28767069

[B36] Li Q. Yang H. Chen Y. Sun H. (2017). Recent progress in the identification of selective butyrylcholinesterase inhibitors for Alzheimer's disease. Eur. J. Med. Chem. 132, 294–309. 10.1016/j.ejmech.2017.03.062 28371641

[B37] Li Q. He S. Chen Y. Feng F. Qu W. Sun H. (2018). Donepezil-based multi-functional cholinesterase inhibitors for treatment of Alzheimer's disease. Eur. J. Med. Chem. 158, 463–477. 10.1016/j.ejmech.2018.09.031 30243151

[B38] Luo X. T. Wang C. M. Liu Y. Huang Z. G. (2015). New multifunctional melatonin-derived benzylpyridinium bromides with potent cholinergic, antioxidant, and neuroprotective properties as innovative drugs for Alzheimer's disease. Eur. J. Med. Chem. 103, 302–311. 10.1016/j.ejmech.2015.08.052 26363866

[B39] Mack A. Robitzki A. (2000). The key role of butyrylcholinesterase during neurogenesis and neural disorders: An antisense-5'butyrylcholinesterase-DNA study. Prog. Neurobiol. 60, 607–628. 10.1016/s0301-0082(99)00047-7 10739090

[B40] Makarian M. Gonzalez M. Salvador S. M. Lorzadeh S. Hudson P. K. Pecic S. (2022). Synthesis, kinetic evaluation and molecular docking studies of donepezil-based acetylcholinesterase inhibitors. J. Mol. Struct. 1247, 131425. 10.1016/j.molstruc.2021.131425 35221376 PMC8881002

[B41] Mangialasche F. Solomon A. Winblad B. Mecocci P. Kivipelto M. (2010). Alzheimer’s disease: Clinical trials and drug development. Lancet Neurol. 9, 702–716. 10.1016/s1474-4422(10)70119-8 20610346

[B42] Matsuzaki K. (2011). formation of toxic amyloid fibrils by amyloid β-protein on ganglioside clusters. Int. J. Alzheimer's. Dis. 2011, 1–7. 10.4061/2011/956104 PMC3034960 21318142

[B43] McGleenon B. M. Dynan K. B. Passmore A. P. (1999). Acetylcholinesterase inhibitors in Alzheimer’s disease. Br. J. Clin. Pharmacol. 48, 471–480. 10.1046/j.1365-2125.1999.00026.x 10583015 PMC2014378

[B44] Mesulam M. M. Guillozet A. Shaw P. Levey A. Duysen E. G. Lockridge O. (2002). Acetylcholinesterase knockouts establish central cholinergic pathways and can use butyrylcholinesterase to hydrolyze acetylcholine. Neuroscience 110, 627–639. 10.1016/s0306-4522(01)00613-3 11934471

[B45] Miao J. Shi R. Li L. Chen F. Zhou Y. Tung Y. C. (2019). Pathological tau from Alzheimer’s brain induces site-specific hyperphosphorylation and SDS- and reducing agent-resistant aggregation of tau *in vivo* . Front. Aging Neurosci. 11, 34. 10.3389/fnagi.2019.00034 30890929 PMC6411797

[B46] Mohsin N. A. Ahmad M. (2020). Donepezil: A review of the recent structural modifications and their impact on anti-alzheimer activity. Braz. J. Pharm. Sci. 56, e18325. 10.1590/s2175-97902019000418325

[B47] Mollazadeh M. Mohammadi-Khanaposhtani M. Zonouzi A. Nadri H. Najafi Z. Larijani B. (2019). New benzyl pyridinium derivatives bearing 2, 4-dioxochroman moiety as potent agents for treatment of Alzheimer’s disease: Design, synthesis, biological evaluation, and docking study. Bioorg. Chem. 87, 506–515. 10.1016/j.bioorg.2019.03.012 30928873

[B48] Mostofi M. Mohammadi Ziarani G. Mahdavi M. Moradi A. Nadri H. Emami S. (2015). Synthesis and structure-activity relationship study of benzofuranbased chalconoids bearing benzylpyridinium moiety as potent acetylcholinesterase inhibitors. Eur. J. Med. Chem. 103, 361–369. 10.1016/j.ejmech.2015.08.061 26363872

[B49] Mushtaq G. Greig N. H. Khan J. A. Kamal M. A. (2014). Status of acetylcholinesterase and butyrylcholinesterase in Alzheimer’s disease and type 2 diabetes mellitus. CNS Neurol. Disord. Drug Targets. 13, 1432–1439. 10.2174/1871527313666141023141545 25345511 PMC5878042

[B50] Musilek K. Komloova M. Zavadova V. Holas O. Hrabinova M. Pohanka M. (2010). Preparation and *in vitro* screening of symmetrical bispyridinium cholinesterase inhibitors bearing different connecting linkage-initial study for Myasthenia gravis implications. Bioorg. Med. Chem. Lett. 20, 1763–1766. 10.1016/j.bmcl.2010.01.034 20138518

[B51] Musilek K. Komloova M. Holas O. Hrabinova M. Pohanka M. Dohnal V. (2011). Preparation and *in vitro* screening of symmetrical bis-isoquinoliniumcholinesterase inhibitors bearing various connecting linkage-implicationsfor early myasthenia gravis treatment. Eur. J. Med. Chem. 46, 811–818. 10.1016/j.ejmech.2010.12.011 21236521

[B52] Nadri H. Pirali-Hamedani M. Shekarchi M. Abdollahi M. Sheibani V. Amanlou M. (2010). Design, synthesis and anticholinesterase activity of a novel seriesof 1-benzyl-4-((6-alkoxy-3-oxobenzofuran-2(3H)-ylidene) methyl) pyridinium derivatives. Bioorg. Med. Chem. 18, 6360–6366. 10.1016/j.bmc.2010.07.012 20673725

[B53] Nadri H. Pirali-Hamedani M. Moradi A. Sakhteman A. Vahidi A. Sheibani V. (2013). 5, 6-Dimethoxybenzofuran-3-one derivatives: A novel series of dual acetylcholinesterase/butyrylcholinesterase inhibitors bearing benzylpyridinium moiety. DARU J. Pharm. Sci. 21, 15. 10.1186/2008-2231-21-15 PMC3599263 23445881

[B54] Nicolet Y. Lockridge O. Masson P. Fontecilla-Camps J. C. Nachon F. (2003). Crystal structure of human butyrylcholinesterase and of its complexes with substrate and products. J. Biol. Chem. 278, 41141–41147. 10.1074/jbc.m210241200 12869558

[B55] Nordberg A. Ballard C. Bullock R. Darreh-Shori T. Somogyi M. (2013). A review of butyrylcholinesterase as a therapeutic target in the treatment of Alzheimer’s disease. Prim. Care Companion CNS Disord. 15, PCC.12r01412. 10.4088/pcc.12r01412 23930233 PMC3733526

[B56] Palin R. Clark J. K. Cowley P. Muir A. W. Pow E. Prosser A. B. (2002). Novel piperidinium and pyridinium agents as water-soluble acetylcholinesterase inhibitors for the reversal of neuromuscular blockade. Bioorg. Med. Chem. Lett. 12, 2569–2572. 10.1016/s0960-894x(02)00483-3 12182862

[B57] Parlar S. Bayraktar G. Tarikogullari A. H. Alptüzün V. Erciyas E. (2016). Synthesis, biological evaluation and molecular docking study of hydrazone-containing pyridinium salts as cholinesterase inhibitors. Chem. Pharm. Bull. 64, 1281–1287. 10.1248/cpb.c16-00221 27581632

[B58] Peng D. Y. Sun Q. Zhu X. L. Lin H. Y. Chen Q. Yu N. X. (2012). Design, synthesis, and bioevaluation of benzamides: novel acetylcholinesterase inhibitors with multi-functions on butylcholinesterase, Aβ aggregation, and β-secretase. Bioorg. Med. Chem. 20, 6739–6750. 10.1016/j.bmc.2012.09.016 23041347

[B59] Picciotto M. R. Higley M. J. Mineur Y. S. (2012). Acetylcholine as a neuromodulator: cholinergic signaling shapes nervous system function and behavior. Neuron 76, 116–129. 10.1016/j.neuron.2012.08.036 23040810 PMC3466476

[B60] Piemonyese L. Tomas D. Hiremathad A. Capriati V. Candeias E. Cardoso S. M. (2018). Donepezil structure-based hybrids as potential multifunctional anti-Alzheimer’s drug candidates. J. Enzyme Inhib. Med. Chem. 33, 1212–1224. 10.1080/14756366.2018.1491564 30160188 PMC6127844

[B61] Prince M. Bryce R. Albanese E. Wimo A. Ribeiro W. Ferri C. P. (2013). The global prevalence of dementia: a systematic review and metaanalysis. Alzheimer's. *&*amp. Dement. 9, 63–75. 10.1016/j.jalz.2012.11.007 23305823

[B62] Rawat A. S. Pande S. Bhatt N. Kharatkar R. Belwal C. Vardhan A. (2013). Synthesis of donepezil hydrochloride via chemoselective hydrogenation. Org. Process Res. Dev. 17, 1617. 10.1021/op400007p

[B63] Rook Y. Schmidtke K. U. Gaube F. Schepmann D. Wunsch B. Heilmann J. (2010). Bivalent β-carbolines as potential multitarget anti-alzheimer agents. J. Med. Chem. 53, 3611–3617. 10.1021/jm1000024 20361801

[B64] Saeedi M. Golipoor M. Mahdavi M. Moradi A. Nadri H. Emami S. (2016). Phthalimide-derived N-benzylpyridinium halides targeting cholinesterases: synthesis and bioactivity of new potential anti-alzheimer’s disease agents. Arch. Pharm. Weinh. 349, 293–301. 10.1002/ardp.201500425 26898241

[B65] Salehi N. Mirjalili B. F. Nadri H. Abdolahi Z. Forootanfar H. Samzadeh-Kermani A. (2019). Synthesis and biological evaluation of new *N*-benzylpyridinium-basedbenzoheterocycles as potential anti-Alzheimer’s agents. Bioorg. Chem. 83, 559–568. 10.1016/j.bioorg.2018.11.010 30471578

[B66] Shafferman A. Kronman C. Flashner Y. Leitner M. Grosfeld H. Ordentlich A. (1992). Mutagenesis of human acetylcholinesterase. Identification of residues involved in catalytic activity and in polypeptide folding. J. Biol. Chem. 267, 17640–17648. 10.1016/s0021-9258(19)37091-7 1517212

[B67] Sharma K. (2019). Cholinesterase inhibitors as Alzheimer's therapeutics (Review). Mol. Med. Rep. 20, 1479–1487. 31257471 10.3892/mmr.2019.10374 PMC6625431

[B68] Shen H. Kihara T. Hongo H. Wu X. Kem W. R. Shimohama S. (2010). Neuroprotection by donepezil against glutamate excitotoxicity involves stimulation of α7 nicotinic receptors and internalization of NMDA receptors. Br. J. Pharmacol. 161, 127–139. 10.1111/j.1476-5381.2010.00894.x 20718745 PMC2962822

[B69] Shi D. H. Huang W. Li C. Wang L. T. Wang S. F. (2013). Synthesis, biological evaluation and molecular modeling of aloe-emodin derivatives as new acetylcholinesterase inhibitors. Bioorg. Med. Chem. 21, 1064–1073. 10.1016/j.bmc.2013.01.015 23380475

[B70] Shuai W. Li W. Yin Y. Yang L. Xu F. Xu S. (2019). Design, synthesis and molecular modeling of isothiochromanone derivatives as acetylcholinesterase inhibitors. Future Med. Chem. 11, 2687–2699. 10.4155/fmc-2019-0125 31596141

[B71] Stanciu G. D. Luca A. Rusu R. N. Bild V. Chiriac S. I. B. Solcan C. (2020). Alzheimer’s disease pharmacotherapy in relation to cholinergic system involvement. Biomolecules 10, 40. 10.3390/biom10010040 PMC7022522 31888102

[B72] Sugimoto H. Ogura H. Arai Y. Iimura Y. Yamanishi Y. (2002). Research and development of donepezil hydrochloride, a new type of acetylcholinesterase inhibitor. Jpn. J. Pharmacol. 89, 7–20. 10.1254/jjp.89.7 12083745

[B73] Taylor P. Radic Z. (1994). The cholinesterases: from genes to proteins. Annu. Rev. Pharmacol. Toxicol. 34, 281–320. 10.1146/annurev.pa.34.040194.001433 8042853

[B74] Tonnies E. Trushina E. (2017). Oxidative stress, synaptic dysfunction, and Alzheimer’s disease. J. Alzheimers Dis. 57, 1105–1121. 10.3233/jad-161088 28059794 PMC5409043

[B75] Vafadarnejad F. Mahdavi M. Karimpour-Razkenari E. Edraki N. Sameem B. Khanavi M. (2018). Design and synthesis of novel coumarin-pyridinium hybrids: *In vitro* cholinesterase inhibitory activity. Bioorg. Chem. 77, 311–319. 10.1016/j.bioorg.2018.01.013 29421707

[B76] Wake R. Araki T. Miyaoka T. Nagahama M. Furuya M. Hayashida M. (2018). Long-term effects of combined treatment with memantine and donepezil on Alzheimer’s disease patients: 72-Week study. Neuropsychiatry (London) 8, 951–960. 10.4172/neuropsychiatry.1000421

[B77] Wang C. Wua Z. Cai H. Xu S. Liu J. Jiang J. (2015). Design, synthesis, biological evaluation and docking study of 4-isochromanone hybrids bearing N-benzyl pyridinium moiety as dual binding site acetylcholinesterase inhibitors. Bioorg. Med. Chem. Lett. 25, 5212–5216. 10.1016/j.bmcl.2015.09.063 26454504

[B78] Wu J. Ishikawa M. Zhang J. Hashimoto K. (2010). Brain imaging of nicotinic receptors in alzheimer's disease. Int. J. Alzheimer's. Dis. 2010, 1–11. 10.4061/2010/548913 PMC3022172 21253523

[B79] Yu L. Cao R. Yi W. Yan Q. Chen Z. Ma L. (2010). Synthesis and binding ability of 1, 2, 3-triazole-based triterpenoid receptors for recognition of Hg(2+) ion. Bioorg. Med. Chem. Lett. 20, 3254–3258. 10.1016/j.bmcl.2010.04.059 20609584

[B80] Yu Q. Holloway H. W. Luo W. Lahiri D. K. Brossi A. Greig N. H. (2010). Long-acting anticholinesterases for myasthenia gravis: synthesis and activities of quaternary phenylcarbamates of neostigmine, pyridostigmine and physostigmine. Bioorg. Med. Chem. 18, 4687–4693. 10.1016/j.bmc.2010.05.022 20627738 PMC2989343

[B81] Zarei S. Shafiei M. Firouzi M. Firoozpour L. Divsalar K. Asadipour A. (2021). Design, synthesis and biological assessment of new 1 -benzyl-4-((4-oxoquinazolin-3(4H)-yl)methyl) pyridin-1-ium derivatives (BOPs) as potential dual inhibitors of acetylcholinesterase and butyrylcholinesterase. Heliyon 7, e06683. 10.1016/j.heliyon.2021.e06683 33869871 PMC8045006

