# Supplementary material for: Recent developments in the design and synthesis of benzylpyridinium salts: Mimicking donepezil hydrochloride in the treatment of Alzheimer’s disease
Source: Front Chem. 2022 Sep 26;10:936240. doi: 10.3389/fchem.2022.936240 (PMC9549744; doi:10.3389/fchem.2022.936240)
Supplement: Supplementary file 1 [file DataSheet1.docx]

**Supplementary Data**

M. Alipour *et al.* [47]:

Synthesis of compounds **5a-s** has shown in Scheme **S1**. First, 2-hydroxybenzaldehydes **1** and ethyl acetoacetate **2** in the presence of catalytic amounts of piperidine prepared 3-acetylcomarin derivatives **3**. In the next step, compound **3** via condensation with pyridine-4-carbaldehyde was converted into the important intermediate **4** under microwave irradiation. The target compounds **5a-s** were synthesized via the addition of compounds **4** to proper benzyl bromide or chloride derivatives in dry acetonitrile under reflux conditions.

**Scheme S1.** Reagents and conditions: (a) piperidine, 50 ^°^C; (b) piperidine, MW irradiation, 30 min; (c) benzyl halide derivatives, CH_3_CN, reflux, 2-4 h.

Based on docking studies of compound **5a** formed a π-cation interaction with Tyr334 and Phe330 that played a significant role in the affinity of the compound at active site and in the inhibitory mechanism. Furthermore, the coumarin and benzyl moieties made the two π-π stacking interactions with Trp84 and Trp279 of CAS and PAS, respectively, and pyridine ring with Phe330 formed a π-π stacking interaction which stabilize orientation of the ligand in the gorge of AChE. The oxygen of coumarin carbonyl group showed a hydrogen bonding interaction with nitrogen of Phe288. Thus, compound **5a** is a dual PAS and CAS binding inhibitor of AChE that is in good agreement with experimental data.

Compound **5o** showed the highest inhibition against BuChE (IC_50_ = 125 nM). It exhibited higher activity than donepezil (IC_50_ = 5380 nM). Moreover, it exposed that anti-BuChE activity of the screened derivatives similar to anti-AChE activity depends largely to the steric and electronic characters of the substituents. Introduction of substitution into 2-, 3- and 4-positions of the benzyl moiety influenced the activity and selectivity (compound **5i** (IC_50_ = 329 nM) and compound **5p** (IC_50_ = 931 nM)). Additionally, the inhibitory activity is very sensitive to the size of the substituents at 2- and 4-positions (**5r** (IC_50_ = 27000 nM) and **5d** (IC_50_ = 15100 nM)) of compounds. It can be observed that the size of the substituent at 3-position is important in order to show considerable activity (**5k**, IC_50_ = 560 nM *vs* **5b**, IC_50_ = 2100 nM). Lastly, incorporation of the second substituent to positions of the benzyl group significantly reduced the activity of the compound (**5k**, IC_50_ = 560 nM) *vs* **5n**, IC_50_ = 5790 nM)) (Fig. **2**).

The same research group [48]:

Initially, combination of 2-hydroxy-3-methoxybenzaldehyde **5** and ethyl acetoacetate **6** and catalytic amounts of piperidine produced 8-methoxy-3-acethyl coumarin **7**. Reaction of compound **7** with pyridine-4-carbaldehyde prepared compound **8**. Finally, the reaction of between intermediate **4** and numerous phenacyl halides in dry acetonitrile yielded derivatives **9a-e** (Scheme **S2**).

**Scheme S2**. Reagents and conditions: (a) piperidine, 50 ^°^C, 1 h; (b) piperidine, MW, 30 min; (c) phenacyl halide derivatives, dry acetonitrile, r.t., 2-3 days.

Resorcinol **10** with chloroacetyl chloride **11** using AlCl_3_ and NaOH provided compound **13**. Thus, compound **13** using ethyl iodide in dry DMF prepared compound **14**. Compound **14** with pyridine-4-carbaldehyde **4** gave the compounds **15**. Lastly, diverse phenacyl halides reacted with compounds **15** in dry acetonitrile to yield the derivaitives **16a-e** (Scheme **S3**).

**Scheme S3.** Reagents and conditions: (a) AlCl_3_, reflux, overnight; (b) NaOH 5%, 0 ^°^C to r.t.; (c) HCl 6 M; (d) ethyl iodide, anhydrous K_2_CO_3_, dry DMF, 80 ^°^C, 2 h; (e) PTSA, reflux, 3 h; (f) 10% sodium hydrogen carbonate, 30-60 min; (g) phenacyl halide derivatives, dry acetonitrile, r.t., 3-4 days.

Based on docking studies, increasing ligand length in coumarin derivatives **9** makes them close to the CAS *via* a π-π interaction, while they interact with gorge via Trp84 residue.

Presence of electron-withdrawing group and slightly lipophilic is accepted at 4-position as seen in compound **9b**. However, electron-donating substituents like methyl (**9c**, IC_50_ = 13.5 µM) and methoxy (**9d**, IC_50_ = 21.0 µM) decreased the activity *via* weakening the critical π-π interaction with Trp84. As the benzyl moiety was oriented near a hydrophilic pocket, the more lipophilic substituents at 4-position were not suitable in lipophilic pocket as in compound **9e** (IC_50_ = 36 µM) having bromo substituent. Therefore, these compounds are dual PAS and CAS binding inhibitor of AChE.

The kinetic study was demonstrating a mixed kind inhibition for the compounds. Furthermore, coumarin derivatives **9** showed weaker activity against BuChE except **9c** (IC_50_ = 8 µM). In the 3-coumaranone series **16**, any group at 4-position of benzyl increases the activity toward **16b** (IC_50_ = 23.5 µM) un-substituent. In this series, compound **16a** (IC_50_ = 15.8 µM) showed the most potent inhibition activity on BuChE. According to the IC_50_ of compounds against BuChE, the order of activity for groups on benzyl moiety was 4-CH_3_ > 4-OCH_3_ > 4-F = 4-H > 4-Br (**9c**, IC_50_ = 8.0 µM, **9d**, IC_50_ = 34 µM, **9a**, IC_50_ = 43 µM, **9b**, IC_50_ = 43 µM, **9e**, IC_50_ = 51 µM). Unlike AChE activity, in this series the order of inhibitory activity was influenced by both the nature and electron-donating of the substituents (Fig. **S1**).

**Fig. S1.** Coumarin and 3-coumaranone derivatives

Khoobi *et al.* [49]:

These compounds were prepared by compounds **20** with diverse benzyl chlorides (Scheme **S4**). Consequently, reaction of 3-pyridine or 4-carbaldehydes **17** with malononitrile **18** and following reaction with 4-hydroxycoumarin with catalytic amounts of magnetic nanocatalyst provided compounds **20**. Thus, compounds **20** reacted with benzyl chlorides bearing electron-donating or electron-withdrawing groups in dry acetonitrile to prepare of compounds **21a-o.**

**Scheme S4.** Reagents and conditions: (a) magnetic catalytic system, H_2_O, 5 min; (b) magnetic catalytic system, H_2_O, 10-30 min; (c) Substituted benzyl chloride, CH_3_CN.

Compound **21e** gave the most potent inhibition for BuChE (IC_50_ = 1.27 µM). It revealed higher activity than donepezil (IC_50_ = 5.38 µM). In 4-pyridinium series, analogues having halide at 4-position displayed enhanced inhibitory activity against BuChE. The presence of two chlorine atoms on the 2- and 4-positions of benzyl group improved the anti-BuChE activity (compound **21f** (IC_50_ = 4.8 µM) *vs* compound **21g** (IC_50_ = 41 µM)). Switch of fluorine at 2- or 3-positions with chlorine improved BuChE inhibition activity (compounds **21g** (IC_50_ = 4.8 µM) *vs* **21b** (IC_50_ = 36 µM), respectively). 3-Pyridinium derivatives against BuChE revealed that 4-fluoro substituent showed good (compound **21j** (IC_50_ = 2.17 µM)). Like the 4-pyridinium series, 2- or 3-chloro compounds **21k** (IC_50_ = 11.2 µM) and **21l** (IC_50_ = 38.4 µM) were more active than 2- or 4-fluoro derivatives **21h** (IC_50_ = 41 µM) and **21i** (IC_50_ = > 50 µM) against BuChE.

Docking study of compound **21a** demonstrated that stabilization of compound in active site is related to an additional π-π interaction which forms between the benzyl group and Trp279. Also, the aromatic moiety in the pyranochromenone fragment made a π-π interaction with Phe330 and Trp84. Furthermore, the amino group of the pyran ring formed an ionic bond with carboxylic side group of Asp72. All derivatives interacted with both regions in the active site (CAS and PAS). Furthermore, the kinetic study reported that compound **21a** is a mixed-type inhibition.

Arab *et al.* [50]:

The reaction of 3-chloropropionic acid, resorcinol **22** and trifluoromethane sulfonic acid provided compound **23**. Intermediate **23** in presence of sodium hydroxid afforded compound **24**. Then, alkyl halide with compound **24** in dry DMF made **25**. The next step, compound **25** was subjected with isonicotinic aldehyde **26** in *p*-toluenesulfonicacid (PTSA) and *N*-alkylation using benzyl bromide derivatives formed of **29a-l** (Scheme **S5**).

**Scheme S5.** Reagents and conditions: (a) 3-Chloropropionic acid, CF_3_SO_3_H, 80 ^°^C, 30 min; (b) 2.0 M NaOH, 5 ^°^C, r.t., 2 h; (c) alkyl halide, K_2_CO_3_, DMF, 80 ^°^C, 3 h; (d) PTSA, toluene, reflux Dean-Stark, 6 h; (e) acetonitrile, reflux, 1-3h.

According to docking studies, compound **29a** can interact with Trp84, Phe330, and Tyr334 of CAS. This compound formed the π-π stacking interactions with Trp84 and Phe330 residues. Moreover, the positively charged nitrogen of compound made a π-cation interaction with Tyr334 which is as main interactions in the CAS. Hydrogen bonding between carbonyl moiety of chroman-4-one and hydroxyl group of Tyr121 accompanied by the hydrophobic interaction between 7-ethoxy group of chroman-4-one and Trp279 was detected in the PAS. Therefore, this compound is a dual PAS and CAS binding inhibitor of AChE. In this study, the anti-BuChE activity of compounds was not evaluated.

Khunnawutmanotham *et al.* [51]:

Scopoletin and 4-(chloromethyl)pyridine hydrochloride interact in the presence of cesium carbonate as a base to provide compound **32**. Compound **32** with numerous alkyl and benzyl halides was changed to the corresponding compounds **33a-o** (Scheme **S6**).

**Scheme S6.** Reagents and conditions: (a) CsCO_3_, DMF, reflux, 3 h; (b) RX, CH_2_Cl_2_, r.t., 24 h or RX, toluene, reflux, 24 h.

Docking studies revealed that compound **33a** formed three π-π interactions: (1) the pyridine ring and Tyr337 of the CAS, (2) the chromene ring and Trp286 of the PAS, and (3) the phenyl ring and Trp86 of the CAS. Also, this compound made two strong hydrogen bonds with the carboxylate group of Glu202. In addition, the hydrogen bond was found between the chromene ring **33a** and the pyridine Tyr124. The fluorine of **33a** was positioned nearby to His447 to form hydrogen bonding interactions. Thus, this compound is a dual PAS and CAS binding inhibitors of AChE. This study did not consider anti-BuChE activity of compounds.

**Fig. S3.** Scopoletin derivatives

Wang *et al.* [52]:

Compound **34** interacted with *tert*-butyl 2-bromoacetate in K_2_CO_3_ and toluene/H_2_O as solvent to give compound **36**, then was hydrolyzed to provide the compound **37**. Compound **37** and *n*-BuLi in THF prepare intermediate **38**. The intermediate **38** reacted with pyridine-4-carboxaldehyde in NaOCH_3_ to afford compound **39**. Final compounds **40a-m** were found via the addition of suitable benzyl bromide analogues to compound **39** in dry acetonitrile (Scheme **S7**).

**Scheme S7.** Reagents and conditions: (a) K_2_CO_3_, H_2_O-toluene, r.t., 30 min; (b) NaOCH_3_, CH_3_OH, r.t. then H_2_O, 95% yield over two steps; (c) *n*-BuLi, THF, -78 ^°^C, 2 h, 50% yield; (d) K_2_CO_3_, CH_3_OH, r.t., 4 h, 60 % yield; (e) CH_3_CN, reflux, 1 h, 60-80% yields

Results from docking study of compound **40a** in the AChE active site displayed that this compound showed a main interaction with Phe330 and Trp84 in CAS region which Phe330 was the important amino acid in compound identification. Phe330 formed a π-cation and π-π interactions with quaternary nitrogen and the pyridinium ring, respectively. Moreover, benzyl moiety showed π-π interaction with Trp84. Furthermore, the 4-isochromanone moiety displayed π-π stacking interaction with Trp279 and a hydrogen bond with Tyr121 in the PAS. Based on docking results, compound **40a** was bonded to PAS and CAS of AChE.

Among compounds, derivative **40a** exhibited the highest inhibitory activity against BuChE (IC_50_ = 2.07 µM). Also, it was more potent than donepezil (IC_50_ = 3.62 µM).

Similar to the AChE inhibition activity, the position and nature of the substituents on the benzyl moiety is important on the BuChE inhibition activity. The presence of chlorine and fluorine groups at the 2- (**40d**, IC_50_ = 3.64 µM) and 4- (**40k**, IC_50_ = 3.56 µM) positions indicated in improving or retaining the activity, however the switch of the halogen atoms on the benzyl group with nitro group strongly diminished the activity (*e.g.* compound **40g** (IC_50_ = 20.92 µM) *vs* compound **40l** (IC_50_ = 29.16 µM)) (Fig. **6**). The presence of methoxy group on benzyl group led to activity almost similar to that of halogen atoms (e.g. **40m** (IC_50_ = 3.06 µM) *vs* **40e** (IC_50_ = 2.44 µM) and **40h** (IC_50_ = 4.33 µM) *vs* **40a** (IC_50_ = 2.07 µM)) (Fig. **S4**). It seems that presence of electron-donating group on benzyl group has negative effect while, electron-withdrawing group has positive effect toward BuChE activity.

Also, compound **40a** indicated the mixed-type inhibition and bind to PSA as well as CAS, which the kinetic study confirmed the docking studies.

Lan *et al.* [53]:

Reaction of starting material compounds **41** and **42** was catalyzed by *p*-CH_3_C_6_H_4_SO_3_H in PhCH_3_ to give the compounds **43**. Then, suitable benzyl bromides **44** with the compounds **43** in dry acetonitrile yielded the benzyl pyridinium bromide salts **45** (Scheme **S8**).

**Scheme S8.** Reagents and conditions: (a) *p*-CH_3_C_6_H_4_SO_3_H, PhCH_3_, 6 h, reflux; (b) CH_3_CN, 1-3 h, reflux.

Compound **45c** showed the most potent activity against BuChE (IC_50_ = 5.4 µM) as similar as donepezil (IC_50_ = 4.5 µM). Donepezil derivatives showed low inhibitor activity against BuChE (IC_50_ > 10 µM), except compound **45c**. It seems that the *N*-benzyl pyridinium fragment is not necessary for the inhibition. Also, diverse electronic feathers of substituents affected BuChE inhibition (compound **45h** (IC_50_ = 12.1%), compound **45a** (IC_50_ = 30.2%) and compound **45g** (IC_50_ = 41.6%) revealed improve in BuChE inhibitory activity (Fig. **S5**).

**Fig. S5.** Inden-1-one bear benzyl pyridinum derivatives

In the docking study of compound **45a**, it made a π-π stacking interaction with Trp84 in the CAS of AChE. Moreover, the 5,6-dimethoxyindanone occupied the PAS by hydrophobic interactions with Trp279 and Gln74 and hydrogen bonds formed between the oxygen of methoxy group and Tyr70. These evidences presented that compound **45a** is bonded to the PAS and CAS of AChE.

The kinetic study of compound **45a**, depicted a mixed-type inhibition for AChE that can bind to the PAS and the CAS regions. Moreover, compound **45a** exhibited inhibitory activity aganist Aβ aggregation (53.7% at 20 µM) and showed also moderate antioxidant activity (0.54 trolox equivalents), low neurotoxicity.

The same research group [54]:

The coumarin derivatives **47** were gave by reaction of resorcinol with ethyl acetoacetate in the presence of a catalytic amounts of concentrated sulfuric acid and 1,4-dioxane. Then, the compounds **47** with the 4-(chloromethyl) pyridine hydrochloride in the presence of K_2_CO_3_ afforded the compounds **48**. In end step, suitable benzyl bromides with the compounds **48** in dry acetonitrile yielded the benzyl pyridinium bromide salts **49a-l** (Scheme **S9**).

**Scheme S9.** Synthesis of coumarin derivatives. Reagents and conditions: (a) H_2_SO_4_, C_2_H_5_OH, 12 h, r.t.; (b) K_2_CO_3_, DMF, 12 h, r.t.; (c) CH_3_CN, 1-3 h, reflux

Docking studies showed that compound **49g** made π-π and cation-π stacking interactions with Trp84 and Phe330, respectively in the CAS. Furthermore, the coumarin moiety formed a hydrogen bond with Arg289 in the PAS. All these observations clearly showed that compound **49g** could bind to the CAS and PAS of AChE.

All compounds showed lower BuChE inhibitory activity. Compound **49d** demonstrated the most potent activity against BuChE (IC_50_ = 0.48 µM) that was 9.5-foled higher than donepezil (IC_50_ = 4.6 µM). But, it was worth noting that 2-position substituted compounds like **49c** (IC_50_ = 1.64 µM), **49b** (IC_50_ = 1.61 µM) and **49d** (IC_50_ = 0.48 µM) showed more potent inhibitory activity (Fig. **4**). It can be deduced that the large size of a 2-position substituent on the benzyl group of the *N*-benzyl pyridinium moiety improved BuChE inhibition.

Kinetic study on the inhibition of ChEs by compound **49g**, showed a mixed-type inhibitor for AChE, able to bind to the PAS and the CAS of AChE. Also, compound **49g** revealed high inhibition against Aβ (62.1%) self-aggregation, and obviously selective inhibition to MAO-B (IC_50_ = 1.57 µM) over MAO-A (IC_50_ = 14.83 µM). Also, compound **49g** was the highest activity against hMAO-B.

Khunnawutmanotham *et al.* [55]:

Derivatives **51** were provided through reaction of salicylaldehyde **50** and *N*-acetylglycine in acetic anhydride. Hydrolysis of **51** with 50% HCl in ethanol afforded 3-aminocoumarin **52**. Reacting **52** with either isonicotinyl chloride (n = 0) or 4-pyridylacetyl chloride (n = 1) made the intermadiates **53**. Finally, the benzylpyridinium bromide salts **54a-i** were obtained by treatment of **53** with substituted benzyl bromides in dichloromethane (Scheme **S10**).

**Scheme S10.** Reagents and conditions: (a) NaOAc, Ac_2_O, 110 ^°^C, 7 h; (b) 50% HCl in CH_3_CH_2_OH, 100 ^°^C, 1 h; (c) (CH_3_CH_2_)_3_CN, CH_2_Cl_2_, r.t., 24 h; (d) PhCH_2_Br, CH_2_Cl_2_, r.t., 72 h

According to docking studies, the most active compound **54k** formed hydrogen bonding interactions with the amino acids: (1) the carbonyl of the amide group and fluorine with the amino acid Trp86, Tyr337, and Phe338 in the CAS; (2) the hydrogens of aryl ring with Glu202 as well as methoxy group with Ser293; (3) the nitrogen of the amide group and dimethoxy substituents on the chromene ring with the amino acid residues Tyr72, Tyr124, Trp286, and Tyr341 in the PAS; (4) the methylene unit of the benzyl group and the amino acid His447; and (5) the carbonyl group of the chromene ring with the acyl pocket amino acid Phe295.

Furthermore, Trp286, Phe338, and Trp86, Tyr72 and Phe338 at PAS and CAS regions made π-π interactions with compound **54k**. Also, the carbonyl groups of the chromene ring of compounds and Phe338 in the CAS and Tyr124 in the PAS were the main interactions in the active site of AChE.

Vafadarnejad *et al.* [56]:

The reaction of compound **57** and pyridin-3-ylmethanamine or pyridin-4-ylmethanamine in the presence of 1-ethyl-3-(3-dimethylaminopropyl)carbodiimide and hydroxybenzotriazole in dry CH_3_CN to obtain compounds **58**. Then, compounds **58** reacted with suitable benzyl halides in acetonitrile to give derivatives **59a-o** (Scheme **S11**).

**Scheme S11.** Synthesis of coumarin-pyridinium hybrids. Reagents and conditions: (a) H_2_O, r.t., 4 h; (b) CH_3_CN, HOBt, EDCl, r.t., 24 h; (c) CH_3_CN, reflux, 3-4 h

Based on docking result, benzyl group of **59a** played a main role in compound identification through π-π stacking interaction with Trp84 and Phe330 amino acid. This interaction was detected for coumarin ring with Trp84. Moreover, pyridinium moiety showed π-π and π-anion stacking interactions withTrp84 and Glu199, respectively.

All screened derivatives exhibited better BuChE inhibitory activity. Compounds **59k** and **59m** bearing chlorine in 2- and 2- and 3-positions on benzyl groups with IC_50_ = 0.32 and 0.43 µM, respectively indicated the best anti-BuChE which both belonging to class I. Compound **59l** bearing chlorine in 2-position on benzyl moiety exhibited good inhibitory activity against BuChE (IC_50_ = 3.69 µM). Compounds **59d**, **59c**, and **59e** in class II established less activity (IC_50_ = 7.19, 29.11, and 26.01 µM, respectively). Compound **59h** having fluorine in 3-position at benzyl pyridinium moiety displayed the highest activity (IC_50_ = 2.78 µM). Furthermore, compounds **59n** and **59o** were established as potent BuChE inhibitors with IC_50_ = 6.45 and 5.92 µM, respectively; while compounds **59f** and **59g** revealed less inhibitory activity (IC_50_ = 17.33 and 24.76 µM, respectively) (Fig. **S6**). The presence of chlorine on benzyl group showed better anti-BuChE activity in the compounds.

**Fig. S6.** Coumarin based benzyl pyridinium derivatives

In case of BuChE, docking studies showed that coumarin moiety of compound **59k** made π-π stacking interaction with Trp231 and Phe329 and benzyl moiety formed π-π stacking interaction with Trp82. In summary, derivatives in group I exhibited higher anti-BuChE activity than derivatives in group II. Moreover, compound **59k** exhibited the most selectivity for BuChE with selectivity index of 101.18.

The kinetic study represented a mixed-type inhibition pattern for compound **59a** against AChE. Similarly, the kinetic study for BuChE inhibitor **59k**, showed a mixed-type inhibihetion pattern.

Hosseini *et al.* [57]:

Reaction of isatoic anhydride **60** and pyridin-3-ylmethanamine **61** or pyridin-4-ylmethanamine **62** in H_2_O to offer compounds **63**. Compounds **63** were changed to compounds **64**. Finally, the compounds **64** reacted with numerous benzyl bromides **65** in acetonitrile to provide the 3 or 4-pyridinium derivatives **66a-q** (Scheme **S12**).

**Scheme S12.** Reagents and conditions: (a) H_2_O, r.t., 8 h; (b) NaNO_2_/H_2_O, HCl, 0 ^°^C, 10 min; (c) CH_3_CN, reflux, 2-3 h

Docking studies showed that the carbonyl group of 4-oxobenzo[*d*]1,2,3-triazin moiety of the most potent compound **66a** made a hydrogen bond with Tyr121 of PAS. This molecule exhibited three π-π interactions with Phe330, Phe331 and Trp84 at CAS. The nitro group in 2-position of benzyl group interacted with catalytic triad residue His440 and oxyanion hole residues Gly118, Gly119, and Ala201.

The 3-pyridinium derivative **66j** having bromine in 4-position of benzyl moiety (IC_50_ = 5.8 μM) exhibited the most potent anti-BuChE activity that was higher donepezil (IC_50_ = 7.2 μM). The other derivatives had lower activity towared donepezil (IC_50_ = 13.7-70.7 μM) (Fig. **S7**).

**Fig. S7.** Coumarin-pyridinium hybrids

In the 3-pyridinium series, introduction of methyl, bromine or nitro in 2-positions on the benzyl group of the compounds **66c** (IC_50_ = 13.7 μM), **66g** (IC_50_ = 16.8 μM) and **66a** (IC_50_ = 17.5 μM) had good effect on inhibitory activity (Fig. **11**). On the other hand, the compound **66b** with an un-substituted benzyl group and fluorine substituent on the 2- and 3-position of the benzyl group, as in the compounds **66b** (IC_50_ = 31.2 μM), **66d** (IC_50_ = 23.9 μM) and **66e** (IC_50_ = 27.8 μM), respectively, decreased anti-BuChE inhibitory activity (Fig. **S7**). The inhibitory activity of the fluorinated analogs had no significant effect on inhibitory activity (*e.g.* **66d**, **66e** and **66f** (IC_50_ = 23.8 μM)) (Fig. **S7**).

In the 4-pyridinium series, the compounds **66m** (IC_50_ = 55.0 μM) and **66k** (IC_50_ = 51.8 μM) with 2-Cl, and 4-NO_2_ substituents on the benzyl ring showed approximately same anti-BuChE potency (Fig. **S7**). The less active compounds among the 4-pyridinium derivatives contained H and 4-F substituents on benzyl ring, derivatives **66l** (IC_50_ = 75.2 μM) and **66p** (IC_50_ = 70.7 μM). The 4-pyridinium series showed lower activity than the 3-pyridinium series (*e.g.* compound **66o** (IC_50_ = 64.6 μM) *vs* compound **66d**) (Fig. **S7**).

According to docking studies, the compound **66j** as the most potent inhibitor of BuChE displayed that the 4-oxobenzo[*d*]1,2,3-triazin moiety interacted with Leu286, Ser198, Phe329 and Trp231 at CAS. The 3-pyridinium moiety made a π-anion interaction with Glu197. Moreover, hydrophobic interactions were made between the bromine in 4-position on benzyl moiety of compound **66j** with amino acids Met437, Trp430, Tyr332, and Ala328 at PAS.

However, the kinetic study of compound **66a** exposed that the compound **66a** inhibited AChE in a mixed-type inhibition style. Therefore, **66a** could bind to both the PAS and CAS regions.

Mollazadeh *et al.* [58]:

Reaction of compound **67** and trimethoxymethane **68** in propanol to offer intermediate **69**. The intermediate **69** reacted with compound **70** in ethanol to give intermediate **71**. Then, derivatives **73a-o** was found from the reaction of compound **71** and numerous benzyl bromides **72** in acetonitrile (Scheme **S13**).

**Scheme S13.** Reagents and conditions: (a) Propanol, reflux, 3 h; (b) Ethanol, reflux, 4 h; (c) CH_3_CN, reflux, 2-3 h

Docking results showed that the most potent compound (**73a**) filled the PAS and the CAS of the AChE active site. The 2,4-dioxochroman moiety made a π-π interaction with the residue Phe331. The carbonyl group at 4-position of 2,4-dioxochroman moiety made three hydrogen bonds with the His440, Ser200 and Gly118 amino acids. Moreover, Trp84 recognized three interactions with the benzyl ring (π-π), pyridinium moiety (π-π), and NH fragment (π-cation) of compound **73a**.

The inhibitory activity of the synthesized compounds against BuChE revealed that compounds **73h** (IC_50_ = 6.43 μM), **73e** (IC_50_ = 15.83 μM), **73j** (IC_50_ = 33.54 μM), **73g** (IC_50_ = 6.18 μM), **73k** (IC_50_ = 5.22 μM), and **73l** (IC_50_ = 7.23 μM) exhibited good to moderate activity whereas the others were inactive compounds (IC_50_ > 100 μM) (Fig. **6**). The chlorine in 3 and 4-positions of derivative **73k**, the most potent compound, showed anti-BuChE activity similar to donepezil (IC_50_ = 5.38 μM).

Additionally, compound **73g** bearing chlorine in 2 and 3-positions was the second most potent compound against BuChE. According to IC_50_ values it can be concluded that the existence of two chlorine on the benzyl ring improved the anti-BuChE activity. Furthermore, compound **73h** having methyl in 2-position and compound **73l** bearing bromine in 3-position, contrasting their 4-substituented (the inactive compounds **73i** (IC_50_ > 100 μM) and **73o** (IC_50_ > 100 μM), respectively) displayed good anti-BuChE activity. Unlike, derivative **73e** bearing fluorine in 3-position indicated more anti-BuChE activity in comparison to compound **73j** bearing fluorine in 4-position (Fig. **6**).

The study of interaction mode of compound **73k** toward BuChE indicated that the 2,4-dioxochroman moiety established two hydrogen bonds with Gly117 and His438. Moreover, this moiety interacted two π-π interactions with the Trp231 and Phe329. In addition, pyridinium ring showed a π-cation and a π-π interactions with His438. Additionally, the benzyl group of the compound made in a π-π interaction with Trp82. The chlorine of benzyl group formed hydrophobic interactions with the Leu125 and Gly121 residues.

In summary, the docking study of compounds **73a** and **73k** displayed interactions with both the PAS and CAS of AChE and BuChE. Also, the kinetic study of compound **73a** exposed a mixed-type inhibition mode against AChE. Moreover, this compound showed a greater self-induced Aβ peptide aggregation inhibitory activity than donepezil and potent neuroprotectivity against H_2_O_2_-induced damage in PC12 cells.

Shuai *et al.* [59]:

As shown in Scheme **S14**, iodination of the compound **74** provided the compound **75**. Then, compound **75** in the presence of K_2_CO_3_ and mercaptoacetate to give compound **77a**. The compound **76** was provided by the reaction of compound **75** with sodium selenide, which was reduced using sodium borohydride in MeOH and reacted with ethyl 2-bromoacetate to offer the compound **77b**. **77a** and **77b** were hydrolyzed to give the corresponding acids **78**, which were then cyclized to give compounds **79**. Then, the key intermediates **79** reacted with pyridine-4-carboxaldehyde in the presence of 10% K_2_CO_3_ solution to provid compounds **80**. Intermediate **81** was synthesized using the same approaches by sodium methoxide. Lastly, derivatives **80** and **81** reacted with diverse benzyl bromide derivatives to produce the final derivatives **82a-l**.

**Scheme S14.** Reagents and conditions. (a) TMSCl, KI, CH_3_CN, r.t., 45 min; (b) Se, NaBH_4_, H_2_O, r.t., 30 min; (c) ethyl 2-mercaptoacetate, K_2_CO_3_, acetone, r.t., 5 h; (d) ethyl 2-bromoacetate, NaBH_4_, CH_3_OH, 3 h; (e) 10% NaOH solution, CH_3_OH, 80 ^◦^C, 30 min; (f1) oxalyl dichloride, DCM, 0 ^◦^C, 30 min; (f2) SnCl_4_, chlorobenzene, r.t., 2 h; (g) isonicotinaldehyde, K_2_CO_3_, H_2_O - *N*,*N*-dimethylformamide, 0 ^°^C, 30 s-2 min; (h) proper benzyl bromide derivatives, CH_3_CN, reflux, 1-2 h; (i1) NaOCH_3_, N-Boc-piperidine-4-carboxaldehyde, THF, r.t., 1-5 min; (i2), CF_3_COOH, DCM, r.t., 2 h.

Docking study showed that the methoxy and carbonyl groups of isothio- and isoselenochromanone moietiy of compounds **82e** and **82f** formed a hydrogen bond with the Phe295 at PAS. Iso- and isothioselenochromanone moieties exhibited a π-π stacking interaction with Trp286 at the PAS. Furthermore, the *N*-benzyl pyridinium moiety of compounds **82e** and **82f** interacted π-π stacking interactions and salt bridge with Trp86 and Asp74, respectively at CAS of AChE. All results revealed that compounds **82e** and **82f** could bind to both the CAS and PAS of AChE, which is in agreement with the results of the kinetic study.

Compound **82e** exhibited low neurotoxicity and moderate antioxidant activity. Furthermore, the docking and kinetic studies exposed that compound **82e** inhibited AChE *via* a mixed-type mode indicating that it could bind to the PAS and CAS of the enzyme.

Rook *et al.* [60]:

Bivalent derivatives **84a-o** of the N2-series were synthesized by the reaction of appropriate β-carbolines **83** with R,ω-dibromoalkanes in DMF as described for bis-pyridinium salts (Scheme **S15**).

**Scheme S15.** Reagents and conditions. (a) 1,6-Dibromohexane, MeCN, r.t.; (b) β-carboline, CH_3_CN

Preparation of β-carboline/pyridine heterobivalent compound **86** and bivalent bis-pyridinium derivative **87** (Scheme **S16**) was achieved through a two-step procedure: ω-bromohexylpyridinium bromide was prepared from pyridine and excessive dibromohexane and then reacted with β-carboline to give **86** (Scheme **S16**).

**Scheme S16.** Reagents and conditions. (a) 1,6-Dibromohexane, CH_3_CN, r.t.; β-carboline, CH_3_CN; (b) 1,6-dibromohexane, DMF, reflux.

Synthesis of the symmetrical N9-bivalent compounds **89a-f** was achieved by reaction of R,ω-dibromoalkane with deprotonated β-carboline **88** in DMSO. **89a-f** were converted to quaternary salts **90a-f** with methyl iodide in acetone. NaBH_4_-reduction of **90b** gave partially reduced β-carboline derivative **91** (Scheme **S17**).

**Scheme S17.** Reagents and conditions. (a) NaOH, 1,6-dibromohexane, DMSO; (b) methyl iodide, acetone, r.t.; (c) NaBH4, methanol

Khorana *et al.* [62]:

Docking study of compound **93k** confirmed formation of a hydrogen bond with Trp84 of AChE. Similar study for **93r** revealed that the quinoline ring constructed a π-π stacking interaction with the Phe330 of AChE.

In a research, [63] 4-hydroxybenzoic acid reacted with 3-aminoethylindole to give the main intermediate **95**. Then, *N*-alkylation of carbazole or indole using 1,4-dibromobutane provided compounds **97** and **99** then reacted with **95** to give compounds **100a-b (**Scheme **S18)**.

**Scheme S18.** Synthesis of compounds **100a** and **100b**. Reagents and conditions: (a) dicyclohexylcarbodiimide, *p*-hydroxybenzoic acid, THF, ice bath; (b), Br(CH_2_)_~~4~~_Br, KOH, DMF; (c) Br(CH_2_)_4_Br, NaOH; TBAB, benzene; (d) K_2_CO_3_, acetone, reflux.

Otherwise, compounds **100c-h** were provided via a two-step process using compound **95** (Scheme **S19)**.

**Scheme S19.** Synthesis of compounds 100c-h. Reagents and conditions: (a) Br(CH_2_)_4_Br, K_2_CO_3_, acetone, reflux; (b) substituted phenol, K_2_CO_3_, acetone, reflux; (c) *N*,*N*-dimethylaminopyridine or quinoline, CH_3_CN, reflux.

Finally, compounds **105a-g** were provided from 4-acetoxybenzoic acid (Scheme **S20)**.

**Scheme S20.** Synthesis of compounds **105a-g**. Reagents and conditions: (a) SOCl_2_, DMF, reflux; (b) o-aminoacetophenone, CH_2_Cl_2_, ice bath; (c) NaOH, MeOH/H_2_O, rt; (d) Br(CH_2_)_n_Br, K_2_CO_3_, acetone, reflux; (e) quinoline, isoquinoline, or pyridine, CH_3_CN, reflux.

Molecular docking study indicated that **100a** (Ki > 10000 nM) favorably interacted with the PAS and the CAS. The indole ring **100a**, the benzene ring and second indole ring made π-π stacking interactions with Try286, Try337 and Trp86, respectively. But, this compound did not made high binding affinity because the π-π stacking interactions in the PAS were not optimized (Fig. **S10**).

**Fig. S10.** Isothio- and isoselenochromanone derivatives

To enhance the stacking interaction with the PAS, compounds **100b-f** were designed in which the indole ring was changed using other hteroaromatic rings like benzophenone and carbazole.

Akrami *et al.* [64]:

In the first stage, compound **106** and pyridin-4-carbalehyde in the presence of *p*-toluenesulfonic acid as a catalyst yielded compound **107**. Then, compound **107** and proper benzyl bromides or chloridse provided compounds **108a-u** (Scheme **S21**).

**Scheme S21.** Reagents and conditions: (a) pyridine-4-carboxaldehyde, *P*-TSA, toluene, reflux (b) benzyl halide derivatives, acetonitrile, 60-70 ^°^C.

Regarding the docking studies, the most active compound **108a** showed three kinds of interactions; π-π, hydrophobic and cation-π interactions were important in the affinity of compound to the amino acids active site. The quaternary nitrogen of the pyridinium ring of compound formed a π-cation interaction with Phe330. Compound also made a π-π interaction with Trp84 in the CAS. Furthermore, the indolinone moiety showed hydrophobic interactions with Tyr70, Tyr121 and Trp279 in the PAS. These main interactions were like those of well-known AChE inhibitors complexed with the enzyme.

The detected IC_50_ values of compounds against BuChE exposed that all derivatives except of **108h** (IC_50_ = 7900 nM) and **108i** (IC_50_ = 7500 nM) were more potent than donepezil (IC_50_ = 5380 nM). Compound **108q** (IC_50_ = 887 nM) showed the highest activity against BuChE which was about 6-folds more than donepezil. Un-substituted compounds (**108d** (IC_50_ = 4300 nM)) showed less activity than substituted benzyl compounds against BuChE. These results exhibited that substitution on the benzyl group had frequently positive effect on anti-BuChE activity. The most potent activity was detected with methyl group in 2-position. However, fluorine in 3-position (compound **108r**, IC_50_ = 4500 nM), methoxy in 4-position (compound **108h**, IC_50_ = 7900 nM) and nitro in 4-position (compound **108i**, IC_50_ = 7500 nM) decreased the inhibitory activity against BuChE (Fig. **9**).

Docking results exposed that the *N*-benzylpyridinium of compound **108a** bound to the CAS and the indoline bound to the PAS of AChE. Kinetics study of compound **108a** confirmed that it inhibited AChE *via* a mixed-type mode.

Luo *et al.* [65]:

The first, the pyridine acids (**110** and **114**) were activated by 1-hydroxybenzotriazole and 1-(3-dimethylaminopropyl)-3-ethylcarbodiimide hydrochloride, and subsequently coupled with tryptamines (**110** or **114**) in the presence of triethylamine in dichloromethane solutions to give the main intermediates (**111** and **115**). Then, the compounds **112a-m** and **116a-f** were provided by the reaction of **111** and **115** with suitable benzyl bromide in dry acetonitrile (Scheme **S22**).

**Scheme S22.** Reagents and conditions: (a) CH_2_Cl_2_, CH_3_CH_2_N, EDCI, HOBT, r.t.; (b) benzyl bromides derivatives, CH_3_CN, reflux, 2-4 h.

Docking results showed that the benzylpyridinium moiety of **116b** interacted via π-cation interactions between the quaternary nitrogen of piperidine ring and Phe330 and Trp84 at the CAS of AChE. Further, the benzyl and pyridinium rings made π-π stacking interactions with Trp84 and Phe330, respectively. Moreover, **116b** interacted π-π stacking interactions between the tryptamine and Trp279 at the PAS. Based on results, compound **116b** was a dual binding site inhibitor of AChE which was in correlation with the kinetic study.

Salehi *et al.* [66]:

In the first phase, compounds **119a-c** were synthesized. Reaction of compound **117b** and pyridin-4-carbalehyde (**118**) in the presence of KMnO_4_/HOAc provided 2-(pyridin-4-yl)benzo[d]oxazole (**119b**). Then, 2-aminothiophenol (**117c**) and pyridin-4-carbalehyde (**118**) were reacted in the presence of Fe_3_O4@NCs-OPO_3_H to make 2-(pyridin-4-yl)benzo[*d*]thiazole (**119c**). Finally, benzyl halides with compounds **119a-c** in dry acetonitrile to yield compounds **120a-q** (Scheme **S23**).

**Scheme S23.** Reagents and conditions: (a) Fe_3_O_4_@nano-cellulose-OPO_3_H, CH_3_CH_2_OH, 50 ^°^C; (b) KMnO_4_/HOAc, r.t., grinding; (c) Fe_3_O_4_@nano-cellulose-OPO_3_H, solvent-free, 100 ^°^C; (d) appropriate benzyl halide, acetonitrile, reﬂux.

The benzyl group of compound **120a** intracted π-π stacking with His439 and Trp83. The pyridinium ring of compound made π-cation and π-stacking interactions with Phe329. Furthermore, the positively charged nitrogen interacted with Asp71 via π-cation interaction.

*In vitro* anti-BuChE activity of compounds was also assayed by the same method. Some compounds showed higher activity than donepezil (IC_50_ =182-2200 nM). Compound **120a** (IC_50_ = 182 nM) was 18-fold more potent than donepezil. Normally, substituted benzyl compounds was less activity than un-substituted **120a** against BuChE. However, 2-chloro, 2-nitro, 2,6-dichloro and 2-chloro-6-fluoro derivatives (**120h** (IC_50_ = 331 nM), **120b** (IC_50_ = 348 nM), **120o** (IC_50_ = 400 nM), and **120r** (IC_50_ = 379 nM), respectively) with IC_50_ ≤400 nM were at least 8-folds more potent than donepezil. All compounds showed higher inhibitory activity against AChE compared with BuChE.

The amyloid-β protein anti-aggregating activity of compounds **120a** and **120c**, as most potent AChE inhibitors, was determined. These compounds revealed a good inhibitory activity on self-induced Aβ aggregation. Compound **120a** (inhibition percentage = 44.9%) was more potent than donepezil and rifampicin (22.0% and 27.5% respectively). The compound **120c** bearing 3-methyl group exhibited less reduction in thioflavin-T fluorescence (28.9%) than un-substituted derivative **120a**.

Baussanne et al. [67]:

The first stage alkylation was achieved in CH_3_CN and the presence of the 2-bromo-*p*-methoxyacetophenone or methyl 2-bromoacetate (alkylating agent) to yield the favorite monoalkylated product. In some cases, the mixture of mono- and bis-alkylated compounds was got that were directly engaged in the next step. To form the indolizine ring, the reaction with methyl propiolate was accomplished in CH_2_Cl_2_ by triethylamine as a base to make the reactive yield. The resulting pyridine-indolizines **125a-d** were isolated and the final alkylation step, with numerous alkylating agents, was finished in CH_3_CN to afford the pyridinium-indolizine salts **126a-h** (Scheme **S24**).

**Scheme S24.** Reagents and conditions: (a) BrCH_2_COR^1^, CH_3_CN, heat; (b) methyl propiolate, N(CH_2_CH_3_)_3_, CH_2_Cl_2_, r.t.; (c) XR^2^, CH_3_CN, heat

Molecular docking studies of compounds **126c** and **126h** exhibited a preserved orientation of their pyridinium moieties within the hBuChE pocket, but a reverse positioning of this group in hAChE: **126c** has its pyridinium group towards the CAS, while **126h** has its pyridinium group toward the arrival of the cavity. Residue Trp286 in hAChE, is in π-stacking interaction with the benzoyl substituent of **126c** and the pyridinium of **126h**.

The **Ind-PyC2** and **Ind-PyC3** hybrids showed some antioxidant activity when tested at 750 μg/mL (up to 95% inhibition of DPPH radical scavenging for 10). In both series, most hybrids were also able to interact with amyloid fibers, even if the inhibitory effect was observed at a high 100 μM concentration.

Saeedi *et al.* [68]:

The reaction of phthalimide **127** or succinimide **129** with 4-(bromomethyl)pyridine **128** in the presence of K_2_CO_3_ in DMF gave compounds **130** or **131**. Following *N*-benzylation of pyridine derivatives **130** or **131** by numerous benzyl halides provided the *N*-benzylpyridinium salts **132a-m** or **133a-c (**Scheme **S25**).

**Scheme S25.** Reagents and conditions: (a) K_2_CO_3_, DMF, 80 ^°^C, 8h; (b) ArCH_2_X, X = Cl, Br, Dry CH_3_CN, reflex, 7-15 h

According to docking studies, compound **132a** made a cation-π interaction with Phe329. This direction permitted creation of a hydrogen bond between fluorine in 2-position and Glu199. Furthermore, the phthalimide moiety of the compound formed a hydrogen bond with Tyr120.

All compounds showed inhibitory activity against BuChE less than AChE. Compounds **132j**, **132h**, **132i**, and **133a** did not display anti-BuChE activity (IC_50_ >100 µM). However, the most potent compound against AChE (compound **132a**) exhibited the highest activity against BuChE (IC_50_ = 8.71 µM). The presence of fluorine or bromine in 2-position of phthalimide moiety improved anti-BuChE activity (*e.g.* compounds **132a** and **132c** (IC_50_ = 5.65 µM)), but other substituents decreased the activity against BuChE. The introduction of fluorine and bromine in 3-positions in the succinimide derivatives had positive effect for anti-BuChE activity (compound **133b** (IC_50_ = 45.61 µM) and compound **133c** (IC_50_ = 50.10 µM)) (Fig. **24**).

Docking study of compound **132a** in the BuChE active site showed that it was well accommodated inside the binding pocket of BuChE through a π-π interaction with phthalimide moiety and Trp82. Furthermore, the fluorobenzyl moiety showed hydrophobic interactions with Trp231 and Phe329.

The kinetic study of compound **132a** showed a mixed-type of inhibition mode.

Nadri *et al.* [70]:

Resorcinol **135** interacted with chloroacetyl chloride in presence of AlCl_3_ provided intermediate **136**. In the next stage, compound **137** was affored by intramolecular cyclization of intermediate **136** with NaOH. The hydroxyl group of compound **137** was alkylated via suitable alkyl halide in dry DMF to prepare compounds **138a-c**. Then, producted compounds reacted with pyridine-4-carboxaldehyde in *p*-toluenesulfonic acid to give compounds **139a-c**. Finally, to compounds **139a-c** was added suitable benzyl bromide or chloride derivatives to yield compounds **140a-u** (Scheme **S26**).

**Scheme S26.** Reagents and conditions: (a) AlCl_3_, reflux, overnight; (b) NaOH 5%, 0 ^°^C to r.t.; (c) HCl 6 M; (d) alkyl halide, anhydrous K_2_CO_3_, 80 ^°^C, 2 h; (e) *p*-TSA, reflux, 3 h; (f) 10% sodium hydrogen carbonate, 30-60 min; (g) benzyl halide derivatives, reflux, 2-3 h.

The same research group [25]:

3,4-Dimethoxyphenol (**141**) interacted with chloroacetonitrile by ZnCl_2_ to produce intermediate of 2-chloro-1-(2-hydroxy-4,5-dimethoxyphenyl) ethane iminium. Next, iminium intermediate and hydrochloric acid yielded compound **142**. Compound **142** and sodium acetate created compound **143**. Reaction of compound **143** and 4-formyl pyridine in the presence of *p*-TSA offered intermediate **144**. Suitable benzyl chloride or bromide derivatives reacted compound **144** in dry acetonitrile to prepare compounds **145a-g** (Scheme **S27**).

**Scheme S27.** Reagents and conditions: (a) *m*-CPBA, CH_2_Cl_2_, reflux 16 h; (b) NaOH 10%, r.t., stir., 4 h; (c) HCl 6 N; (d) ClCH_2_CN, HCl gas, ZnCl_2_, 0 ^°^C, 2.5 h, stir.; (e) HCl 1 N, reflux, 90 min; (f) Sodium acetate trihydrate, ethanol, reflux, 10 min; (g) Pyridine-4-carboxaldehyde, *p*-TSA, toluene, reflux; (h) Substituted benzyl halide, CH_3_CN, reflux.

According to docking study of compound **145e**, the phenyl ring of benzofuranone and Trp279 formed a π-π stacking at the PAS. Also, a hydrogen bond was made between the 6-methoxy and the Tyr70. Furthermore, the benzyl pyridinium moiety of the compound showed hydrophobic interactions with Phe330, Trp84 and Glu199 at the CAS. π-π Stacking and a π-cation interactions also was formed between bezyl pyridinium and Trp84 and Phe330, respectively.

The compound **145f** (IC_50_ = 740 nM) having fluorine in 3-position revealed the most potent activity against BuChE. Shifting the fluorine from 3- to either 2- or 4-position of reduced the activity (compounds **145e** and **145g** (IC_50_s = 1620 and 960 nM, respectivley)). The precence of electron-donating group on the benzyl ring decreased activity. In addition, changing the position of methyl (**145a**) from 2- to either 3- (**145b**) or 4- (**145c**) position significantly diminished activity (IC_50_s = 3620, 5310 and 7600 nM, respectivley) against BuChE. Fluorinated compounds showed better anti-BuChE activity than compounds having methyl group (*e.g.* compound **145f** *vs*. compound **145b**). All compounds except compounds **145b** and **145c** showed higher anti-BuChE activity than donepezil (IC_50_ = 5400 nM)) (Fig. **S13**).

**Fig S13.** Indole, β-carboline and quinoline derivatives

Baharloo *et al.* [71]:

Salicylaldehyde derivatives **146** with 4-(bromomethyl) pyridine **147** interacted in K_2_CO_3_ to creat O-substituted salicylaldehyde derivatives **148**. Cyclization of derivaitves **148** by *t*-BuOK in DMF provided derivatives **149**. Then, derivatives **149** were benzylated with suitable benzyl bromide to afford the final compounds **150a-o** (Scheme **S28)**.

**Scheme S28.** Reagents and conditions: (a) K_2_CO_3_, DMF, 80 ^°^C; (b) *t*-BuOK, DMF, 80 ^°^C; (c) CH_3_CN

The benzyl pyridinium moiety of the compound **150b** positioned nearby Trp84. The positively charged nitrogen formed π-cation interaction with Phe330 and Tyr334. Furthermore, pyridinium ring made a π-π stacking with Tyr121. Another π-π stacking interacted between benzofuran moiety and Trp279 in the PAS.

Mostofi *et al.* [72]:

Salicylaldehyde derivatives **151** with chloroacetone (**152**) interacted in K_2_CO_3_ to provide 2- acetylbenzofurans **153**. Reaction of compounds **153** with either pyridine-3-carboxaldehyde or pyridine-4-carboxaldehyde in *n*-butanol prepared un-saturated ketones **154** or **155**, respectively. Next, compounds **154** or **155** with suitable benzyl halide created compounds **156a-v** (Scheme **S29**).

**Scheme S29.** Reagents and conditions: (a) K_2_CO_3_, dry acetone; (b), (c) Piperidine, *n*-Butanol, MW, 150 ^°^C, 30 min; (d), (e) Approperiate benzyl halide, CH_3_CN, reflux, 2-4 h

According to docking studies, Trp83 amino acid of CAS was interacted to formation of π-π stacking with benzofuran ring compound **156c**. Likewise, the bromophenyl of the compound was found with Trp278 in the π-π stacking at the PAS. Whereas the benzyl ring was stacked with Trp278 and bromine in 2-position interacted with Arg288, respectively. The pyridinium ring formed a π-cation interaction with Tyr333.

Abedinifar *et al.* [73]:

The salicylaldehyde derivatives **157** and ethyl bromoacetate in K_2_CO_3_ gave ethyl benzofuran-2-carboxylates. Following hydrolysis in aqueous ethanol/KOH provided **159**. The amidation reaction of **159** with 3-(methylamino) pyridine or 4-(methylamino) pyridine in the presence of hydroxybenzotriazole and *N*-(3-dimethylaminopropyl)-N′-ethylcarbodiimide hydrochloride as coupling agents resulted in **160** and **161**. The *N*-benzylation of the latter compounds with suitable benzyl halides in acetonitrile prepared comounds **162a-o** (Scheme **S30**).

**Scheme S30.** Reagents and conditions: (a) Ethyl bromoacetate, K_2_CO_3_, Dry DMF, 90 ^°^C, 4–6 h; (b) KOH, Ethanol: H_2_O (2:1), reflux; (c) (Pyridin-3-yl)methanamine or (pyridin-4-yl)methanamine; HOBT, EDC.HCl, Dry CH_3_CN, r.t., 24–48 h; (d) substituted benzyl halides, CH_3_CN, reflux, 2-4 h.

Docking studies indicated four cation-π interactions between quaternary nitrogen of pyridine ring of compound and Trp86 and Tyr337. Furthermore, benzofuran moiety of the compound interacted in a π-π interaction with Trp286 and Tyr341. This compound formed two hydrogen bonds Asp74.

In the case of anti-BuChE activity, **162f** showed the most potent activity, 100-fold stronger (IC_50_ = 5.4 µM) than donepezil (IC_50_ value of 0.054 μM). The 2- and 3-substituted derivatives exhibit better inhibition toward BuChE than the 4-substituted ones (*e.g.* compound **162b** (IC_50_ = 0.45 µM) and compound **162c** (IC_50_ = 0.11 µM) *vs.* compound **162d** (IC_50_ = 0.65 µM)).

The introduction of methoxy in 7-position of benzofuran led to the decrease BuChE inhibitory activity (**162g** (*e.g.* compound **162b** (IC_50_ = 2.1 µM) and compound **162c** (IC_50_ = 13.8 µM) *vs.* compound **162d** *vs*. **162h** (IC_50_ = 0.87 µM)) (Fig. **S14**).

**Fig. S14.** Indole-alkoxybenzamide derivatives

It was also found that compounds **162f** and **162b** were good inhibitors against Aβ-fibrillization (33.1 and 46.4% inhibition, respectively) towared donepezil (22%) and rifampicin (27.5%).

Musilek *et al.* [74]:

Regarding AChE, the best scored docking pose of **163l** (-7.61 kcal/mol) presented seeming interactions with the CAS and PAS amino acids. Namely, pyridinium moiety of compound formed a strong π-π or cation-π interaction with Trp86 and Trp286 at the PAS. Likewise, compound **163v** (-9.67 kcal/mol) showed double T-stacking with Trp86 and Trp286. Compounds **163l** and **163v** exhibited the same interactions and therefore caused in the *in vitro* screening with the same IC_50_ = 0.4 and 0.2 µM, respectively (Fig. **S15**).

**Fig. S15.** Benzophenone derivatives

Compound **163v** (IC_50_ = 0.8 µM) revealed the highest activity against BuChE, which is comparable to standard compound **163b** (IC_50_ = 0.8 µM) (Fig. **S15**).

For anti-BuChE activity, top-scored docking pose of **163l** (-6.91 kcal/mol) displayed the π-π or cation-π interaction with Trp82 and the T-stacking with Trp231. Likewise, compound **163v** (-7.45 kcal/mol) showed π-π or cation-π interactions with Trp82 and T-stacking with Trp231. Furthermore, naphtylene spacer showed one T-stacking with Phe329. This interaction may clarify, that the lower flexible spacer (naphtylene) in compound **163v** showed higher activity against BuChE (0.8 µM *vs* 5 µM of **163l**) (Fig. **S15**).

The kinetic studies of two most promising compounds confirmed non-competitive inhibition of AChE.

**Scheme S31.** Prepared bisquaternary pyridinium salts bearing different linkers.

Similar research group [75]:

Concerning the AChE, compound **164h** (-11.09 kcal/mol) showed π-π or cation-π interactions between both isoquinolinium parts and Trp286. Isoquinolinium part also exhibited hydrophobic interactions with Tyr72, Tyr124, Trp286 and Phe297.

Regarding BuChE, compound **164h** presented π-π or cation-π interaction with Trp82 and the T-stacking with Trp231. Furthermore, one isoquinolinium ring showed additional T-stacking with His438, Phe329 and Phe398.

The isoquinolinium part was vital which was led to π-π and cation-π interactions with amino acids of ChE. Additionally, the isoquinolinium compounds were established as the highest activity against hAChE towared previous pyridinium ones.

**Scheme S32.** Preparation of bis-isoquinolinium salts.

This group [75]:

Similar to hAChE results, some derivatives (**165a-e** (IC_50_s = 34, 20, 53, 5.5 and 5.1 µM, respectively), **165m-s** (IC_50_s = 75, 241, 19, 4.4, 2.4, 1.9 and 1.3 µM, respectively)) showed BuChE inhibition. Furthermore, compounds **165f-l** (IC_50_s = 0.6, 0.7, 0.016, 0.15, 0.06, 0.11 and 0.019 µM, respectively) resulted in potent hBuChE inhibitory activity. Among hBuChE inhibitors, compounds **165f-l** and **165t** (IC_50_ = 0.12 µM) exceeded the commercial standard ethopropazine (Fig. **S22**).

**Fig. S22.** Bis-pyridinium derivatives

Both selected compounds **165h** and **165t** were non-competitive inhibitors of hAChE with almost no influence on the substrate hydrolysis (acetylthiocholine). Prepared compounds did not show higher selectivity than compound **165g** (IC_50_ = 0.016 µM).

According to docking studies, the most potent compound **165h** showed top-scored docking pose. Pyridinium ring interacted with Tyr124, Phe297, Tyr337 and Phe338, while its 4-*tert*-butyl part formed CH-π interactions with Trp86, Tyr337 and His447. Furthermore, pyridinium ring made cation-π interaction with Tyr286 and its 4-*tert*-butyl part showed CH-CH interactions with Leu289 and Glu292.

Docking study of the most promising compound **165h** for hBuChE indicated cation-π interactions between one pyridinium ring with Tyr332 and Phe329, whereas its 4-*tert*-butyl part interacted through CH-π interactions with Trp82 and His438. The second pyridinium ring exhibited cation-π interaction with Phe329 and its 4-*tert*-butyl part made CH-π interactions with Trp231 and Phe398.

Results from *in vitro* assay and docking study indicate that the pyridinium fragment was very important in making π-cationic interaction.

**Scheme S33.** Preparation of SAD-128 analogues

Komloova *et al.* [76]:

The kinetic study established that compounds **168c** and **168d** were bonded to PAS and CAS of the AChE. This assay established non-competitive character of the inhibition for both compounds **168c** and **168d**.

Based on docking results, pyridinium ring of compounds **168c** and **168d** indicated cation-π interaction with Trp86. Quinolinium part interaced with Trp286 and Tyr124 residues at the PAS.

Each compound with even number of methylene groups in the spacer was slightly lower activity than the odd number. Compounds having 9 methylene group (**168a,** IC_50_ = 0.35 µM) and 10 methylene group (**168b,** IC_50_ = 0.37 µM) in the spacer revealed the most potent activity against BuChE edrophonium (IC_50_ = 1370 µM) and **BW284C51** (IC_50_ = 354 µM). In addition, in the oquinolinium-pyridinium group, compounds with 11 methylene unit (**168e,** IC_50_ = 0.9 µM) and 12 methylene group (**168d,** IC_50_ = 0.68 µM) in the spacer indicated the most potent activity against BuChE (Fig. **S18**).

**Fig. S18.** Benzofuran-based *N*-benzylpyridinium derivatives

**Scheme S34.** Reagents and conditions: (a) Br(CH_2_)_n_Br, MeCN, 60 ^°^C; (b) Pyridine, DMF, 70 ^°^C

Parlar *et al.* [77]:

In the first step, hydrazine reacted with 4-chloropyridine to give 4-hydrazinylpyridine, next 4-hydrazi-nylpyridine with aromatic ketones in ethanol to provide derivatives **169**. Finally, compounds **169** were quaternized with alkyl halides to prepare the compounds **170a-I** (Scheme **S35**).

**Scheme S35.** Reagents and conditions: (a) RCOCH_3_; (b) R'-Br

Based on docking, compound **170e** showed three main binding interactions in AChE active site. Hydrazone group formed a hydrogen bond with Phe330 residue. Moreover, pyridinium ring interacted π-π interactions with Tyr334 and Trp279 residues. Based on docking results, compound **170e** can interact with both the PAS and CAS and thus this compound can be speculated to have a dual binding potential.

Compound **170d** exhibited the highest activity against BuChE (IC_50_ = 0.95 μM). All of the compounds except **170c** exhibited selectivity toward AChE or BuChE enzyme. In addition, compounds having phenylpropyl moiety had more the selectivity tendency than toward AChE in compared with BuChE while, compounds bearing phenoxyethyl showed more the selectivity tendency than toward BuChE in compared with AChE.

Shi *et al.* [78]:

The docking study of compound **171a** in AChE revealed that the OH in 8-position of the compound could interact with HIS440 in the CAS of the AChE through the hydrogen bonding interaction. This also proved the importance of the hydroxyl of aloe-emodin derivatives for the AChE-inhibition activity. The pyridine ring of the compound could interact with the Trp279 and Tyr334 in the PAS through the π-π interaction. Binding of **171a** was energetically favorable, with binding energy of -9.04 kcal/mol.

The kinetic study and the molecule modeling confirmed that the compound **171a** could bind to the CAS and PAS at the same time. Compound **171a** might prevent the aggregation of Aβ induced by AChE based on its AChE-inhibition activity.

Lan *et al.* [11]:

In first, hydrazine reacted with 4-chloropyridine to give 4-hydrazinylpyridine next 4-hydrazinylpyridine with aromatic ketones in ethanol provided derivatives **172-174**. Finally, the derivatives **172-174** with alkyl halides in ethanol afforded final compounds **176a-n** (Scheme **S36**).

**Scheme S36.** Reagents and conditions: (a) DMAP/EDCI, CH_2_Cl_2_, r.t., 12 h; (b) CH_3_CN, reflux, 1-3 h.

Kinetic study showed that compound **176a** is a mixed-type inhibitor against AChE and can be bind to the CAS and the PAS of AChE. Also, docking study demonstrated that the benzyl pyridinium part of compound **176a** made π-π stacking interactions with Trp84 and Phe330. Besides, the charged nitrogen of the pyridine ring at compound **176a** showed cation-π interaction with Trp84 and Phe330 at the CAS. The cinnamic acid moiety interacted with Trp279 and Gln74 at the PAS. Thus, based on results compound **176a** can bind to the PAS and CAS of AChE.

In addition, compound **176a** displayed metal-chelating capacity, inhibition of Aβ aggregation and inhibition of Cu^2+^-induced Aβ aggregation. Compound **176a** also exhibited a neuroprotective effect against Aβ toxicity in PC12 cells and was established to penetrate into brain by the PAMPA-BBB assay.

Ghotbi *et al.* [79]:

Compound **179** reacted with pyridin-4-ylmethanamine **180a** or pyridin-3-ylmethanamine **180b** in EDCl and HOBt in dry CH_3_CN to prepare compounds **181**. Next, compounds **181** reacted with suitable benzyl halides **182** in acetonitrile to give compounds **183a-u** (Scheme **S37**).

**Scheme S37.** Reagents and conditions: (a) THF, reflux, 5 h; (b) CH_3_CN, HOBt, EDCl, r.t., 24 h; (c) CH_3_CN, reflux, 2-3 h

Docking study of compound **183a** indicated that benzyl part played a key role in compound identification through π-π stacking interaction with Trp86. Moreover, pyridinium part made π-π stacking or cation-π interactions with Tyr337, Phe338, and Tyr341. Additionally, the thiazole ring made π-π stacking interaction with Tyr72. Compound **183a** formed a hydrogen bond with Phe295 residue. This compound also displayed hydrophobic interactions with Trp86, Trp286, Tyr337, and Tyr341 residues. It seems that **183a** made main interactions with both CAS and PAS parts of the AChE.

Kinetic studies of compounds **183a** and **183b** confirmed a mixed-type of AChE inhibition mechanism.

Synthesized compounds demonstrated much weaker inhibitory effects on BuChE with the IC_50_ = 25.40 μM detected for compound **183i**. All compounds except **183i**, **183j**, **183h**, **183b** and **183n** showed no activity against BuChE (IC_50_ > 100 µM). The substitution of more electron-withdrawing groups at 4-position reduced the inhibitory activities of 4-pyridinium compounds against BuChE (*e.g.* compound **183i** (IC_50_ = 25.40 µM) *vs*. compound **183j** (IC_50_ = 56.54 µM)) (Fig. **S19**).

**Fig. S19.** Benzofuran-2-carboxamide-*N*-benzyl pyridinium derivatives

Furthermore, **183a** and **183b** displayed anti-β-amyloid self-aggregation activities (20.38 and 42.66% respectively) higher than donepezil (14.70%) (Fig. **S19**). Besides, compounds **183b** and **183k** revealed effective neuroprotective agents in H_2_O_2_-induced oxidative stress on PC12 cells almost similar to those observed for donepezil.

Abdullaha *et al.* [80]:

The first step is the activation of the aromatic acids with EDC followed by coupling with amines in dry solvent. Finally, refluxing pyridine amide intermediates with different benzyl halides/alkyl halides in acetonitrile yielded the corresponding pyridinium benzamides **191x-af**. To probe the role of the positive charge on nitrogen, the pyridine ring was replaced with a piperidine ring (**193a-b**). Further, the naphthalene ring was also replaced with different biphenyls such as phenoxy phenyl, biphenyl, benzoyloxy phenyl, phenoxy benzyl (**191aq-ar**) to explore the role of naphthalene that increases the potency against BuChE. Furthermore, to check the role of benzyl ring in the activity, it was replaced with ethyl moiety (**191au**). The treatment of aryl carboxylic acids **184** with oxalyl chloride gave the corresponding acid chlorides **185**. The reaction of acid chlorides **185** with 4-aminopyridine produced pyridinyl benzamides **186**. Finally, refluxing the pyridine benzamides **186** in the presence of benzyl bromide in acetonitrile resulted in the formation of pyridinium compounds **187a-w** (Schemes **S38** and **S39**).

**Scheme S38.** Reagents and conditions: (a) oxalyl chloride, Dry THF, N_2_, 0 ^°^C, 2h; (b) 4-aminopyridine, dry THF, 0 ^°^C, 10 min; (c) acetonitrile, benzyl bromide, 80 ^°^C, 1h.

**Scheme S39.** Synthesis of naphthyl substituted pyridinium benzamides **191x-af, 7a-b, 4aq-at, 4au**. Reagents and conditions: (a) EDC.HCl, (CH_3_CH_2_)_3_N, Dry DCM, N_2_, r.t., 3h; (b) acetonitrile, substituted benzyl halides/alkyl halides, 80 ^°^C, 1h.

The significant difference in the BuChE inhibition activity of compound **187g** (Not determined), **187w** (IC_50_ = 1.591 µM), and all other compounds, could be attributed to the presence of bicyclic ring in **187w** and **187g**. Compound **187w** was further optimized by substituting different electron-withdrawing and electron-donating groups on *N*-benzyl moiety (series A) and also incorporating a methyl linker (**191ag** (IC_50_ = 2.41 µM)) and an ethyl linker (**191ag-ap**) between amide and pyridine ring (series B). Initially, the naphthyl portion was kept constant, and modifications were imparted at other parts of the structure.

Molecular docking studies demonstrated that the compound **193b** accommodated in the active site gorge interacting with the amino acids of the PAS and CAS of the both enzymes. The naphthalene moiety of **193b** oriented toward the peripheral site and its *N*-benzyl group toward the catalytic site of the AChE active site gorge. The close packing of the molecule inside the active site gorge was observed by virtue of its various types of interactions such as H-bonding, π-π stacking, and π-cation interactions with residues of active site gorge. Further, it attained a position wherein carbonyl moiety establishes H-bonding with the Phe295 and Arg296 and -NH of amide forming an H-bonding with Asp74 at the lining of the gorge. In the CAS of active site gorge, the piperidine nitrogen has reproduced the cation-π interaction with Tyr337 residue. The piperidine nitrogen also displayed cation-π interaction with Trp86 of the CAS. The *N*-benzyl ring and naphthalene moiety showed π-π interactions with Trp86, and Trp286 residues, respectively indicating that the compound **193b** has ability to interact with both sites of the AChE active site gorge. Additionally, the NH of amide linker formed an H-bonding with Asp74, which is present at the lining of the gorge. Further, the docking study was also carried of **193b** with BuChE. The NH of amide moiety was found to be engaged in H-bonding interaction with Asp70, and the nitrogen of piperidine displayed a cation-π interaction with Trp82, and benzyl attained a position to stack with the Trp82. Docking results for the inhibition of BuChE by **193b** depicted that the molecule bends in a U-shaped format because of the available space within the cavity.

Kinetic analysis was found that the increase in the inhibitor concentration results in a decrease in the V_max_ leaving the K_m_ value constant, indicating that the compound **193b** is a non-competitive inhibitor of AChE. Further for eqBuChE has shown that with an increase in the inhibitor concentration has led to a decrease in the V_max_ and an increase in K_m_ value, indicating that the compound **193b** is a mixed inhibitor of BuChE.

To investigate the multi-targeted nature of the identified dual AChE/BuChE inhibitor, its ability to inhibit Aβ-aggregation was also studied. Following thioflavin-T based fluorometric assay, the inhibitory activity of **193b** was determined against self-induced Aβ42 aggregation. Compound **193b** depicted Aβ42 anti-aggregating activity of 25% at 10 mM.

Zarei et al. [81]:

Briefly, the reaction of 3-hydroxy-4-methoxy benzoic acid (**194**) with iodomethane in DMF furnished methyl 3,4-dimethoxy benzoat (**195**). Nitration and subsequent reduction of compound **195** afforded compound **197**. The 6,7-dimethoxyquinazolin-4(3H)-one (**198**) was prepared by ring closure of **197** in the presence of formamidine acetate. The intermediates **198** or **201** reacted with 3 or 4-(chloromethyl)pyridine in DMF in the presence of K_2_CO_3_ to obtain compounds **199a**,**b** and **202a**,**b**, respectively. Final compounds **203a-l** were gained by adding appropriate benzyl halide derivatives to complexes **199a**,**b** and **202a**,**b** in refluxing dry acetonitrile (Scheme **S40**).

**Scheme S40.** Reagents and conditions: (a) CH_3_I, DMF, r.t., overnight; (b) HNO_3_; (c) SnCl_2_, HCl, 0 ^°^C to r.t., 3h; (d) formamidine acetate, DMF, 100 ^°^C, 16h; (e) 3 & 4-chloromethyl pyridine, K_2_CO_3_, DMF, 50 ^°^C, 4h; (f) benzyl halide derivatives, CH_3_CN, reflux, 3-5 h.

According to the interaction mode of **203a**, the nitrogen of quinazolinon aligned toward Ser122 residues *via* an H-bonding interaction and oxygen of carbonyl group interacted with Gly123 through an H-bonding interaction. In case of compound **203h**, nitrogen of quinazolinon aligned toward Tyr130 residues *via* an H-bonding interaction and methoxy groups interacted with Gly123 and Ser124 through H-bonding interactions. These tree key H-bonding interactions demonstrated the high inhibitory potency of compound **203h** for AChE.

For BuChE inhibitory activities, compounds in first group revealed similar manner to the AChE inhibitory activity and compound **203c** with fluorine at 4-position showed better result in comparison to its AChE inhibition activity (IC_50_ > 100 μM for AChE, IC_50_ = 70.43 μM for BuChE). For **203e** with un-substitution on benzyl ring, BuChE inhibition reached the IC_50_ value of 12.63 μM, lower compared with AChE which enjoys an IC_50_ of 41.21 μM. In the first group, the BuChE inhibitory activities were more potent than AChE inhibition except for **203a** with slightly better AChE activity (IC_50_ = 5.90 μM for AChE, IC_50_ = 6.76 μM for BuChE) that was the best inhibitor in this group. For compounds **203f** to **203l** lower inhibitory effect on BuChE was observed (except **203f** and **203j** with intermediate IC_50_ values of 8.77 μM, 18.84 μM respectively) (Fig. **13**).

For compound **203a** in complex with BuChE, a conventional H-bonding interaction of carbonyl oxygen with His438 and also π-π stacking interactions for phenyl ring of quinazolinon and benzyl ring with several active site amino acids were observed. Docking study of compound **203h** as a selective inhibitor of AChE was performed in the active site of BuChE. There were no major interactions with the active site residues in comparison to AChE binding pattern especially lacking H-bonding interactions that is in line with *in vitro* assay for compound **203h** as a weak BuChE inhibitor.

Hassanzadeh *et al.* [82]:

**Scheme S41.** Reagents and conditions: (a) K_2_CO_3_, glacial acetic acid, reflux, 8h; (b) EDCI, HOBT, chloroform, r.t., 15-24h; (c) KI, acetonitrile, 80 ^°^C, overnight.

Docking studies have shown that compounds **210a** and **210f** were bound to both the CAS and PAS at the same time thus, providing an explanation for its potent inhibitory activity against AChE. The phthalimide moiety was engaged in π-π stacking interaction with Trp279 located in the PAS of the AChE. Also, the carbonyl group of phthalimide ring formed a H-bonding with Tyr121. The pyridinium moiety created a π-π stacking with Phe330 while the charged nitrogen of the same moiety also formed cation-π interactions with Trp84. The benzyl moiety was oriented toward Trp84 in the CAS through π-π stacking interaction and played a vital role in ligand recognition in this site of the enzyme. However, it should be noted that the fluorine atom at the 4-position of the benzyl moiety created two H-bonds with Gly117. Additionally, in the case of compound **210f**, the fluorine formed another hydrogen bond with the hydroxyl group of Tyr130. Moreover, hydrophobic interactions could be observed between **210a** and aromatic residues of Phe330 in the middle of the active gorge.

**Fig. S2.** 5-Oxo-4,5-dihydropyrano[3,2-*c*]chromenes linked to *N*-benzylpyridinium derivatives

**Fig. S4.** 4-Isochromanone derivatives

**Fig. S8.** Triazin-pyridinium hybrids

**Fig. S9.** Benzyl pyridinium derivatives bearing 2,4-dioxochroman moiety

**Fig. S11.** β-Carboline scaffolds

**Fig S12.** *N*-Monophenylcarbamate analogues of neostigmine methyl

**Fig. S15.** melatonin-derived benzylpyridinium derivatives

**Fig. S16.** 5,6-Dimethoxybenzothiophene scaffold

**Fig. S17.** Benzofuranone-based derivatives

**Fig. S20.** Bispyridinium hybrids

**Fig. S21.** Bis-isoquinolinium derivatives

**Fig. S23.** Isoquinolinium-pyridinium and quinolinium-pyridinium bisquaternary compounds, edrophonium and BW284C51

**Fig. 24.** Pyridinium bearing alkylphenyl derivatives

**Fig. S25.** Compounds containing thiazole and pyridinium moieties

**Fig. S26.** Compounds bearing pyridinium benzamides moiety
